# Identification of early pericyte loss and vascular amyloidosis in Alzheimer’s disease retina

**DOI:** 10.1007/s00401-020-02134-w

**Published:** 2020-02-10

**Authors:** Haoshen Shi, Yosef Koronyo, Altan Rentsendorj, Giovanna C. Regis, Julia Sheyn, Dieu-Trang Fuchs, Andrei A. Kramerov, Alexander V. Ljubimov, Oana M. Dumitrascu, Anthony R. Rodriguez, Ernesto Barron, David R. Hinton, Keith L. Black, Carol A. Miller, Nazanin Mirzaei, Maya Koronyo-Hamaoui

**Affiliations:** 1 Department of Neurosurgery, Maxine Dunitz Neurosurgical Research Institute, Cedars-Sinai Medical Center, 127 S. San Vicente Blvd., Los Angeles, CA 90048 USA; 2 Department of Biomedical Sciences and Eye Program, Board of Governors Regenerative Medicine Institute, Cedars-Sinai Medical Center, Los Angeles, CA USA; 3 Department of Biomedical Sciences, Division of Applied Cell Biology and Physiology, Cedars-Sinai Medical Center, Los Angeles, CA USA; 4 Department of Neurology, Cedars-Sinai Medical Center, Los Angeles, CA USA; 5 Norris Comprehensive Cancer Center, Keck School of Medicine, University of Southern California, Los Angeles, CA USA; 6 Doheny Eye Institute, Los Angeles, CA USA; 7 Departments of Pathology and Ophthalmology, Keck School of Medicine, USC Roski Eye Institute, University of Southern California, Los Angeles, CA USA; 8 Department of Pathology Program in Neuroscience, Keck School of Medicine, University of Southern California, Los Angeles, CA USA

**Keywords:** Vascular damage, Neurodegeneration, Pericytes, Cerebral amyloid angiopathy, Retinopathy, Alzheimer’s disease

## Abstract

Pericyte loss and deficient vascular platelet-derived growth factor receptor-β (PDGFRβ) signaling are prominent features of the blood–brain barrier breakdown described in Alzheimer’s disease (AD) that can predict cognitive decline yet have never been studied in the retina. Recent reports using noninvasive retinal amyloid imaging, optical coherence tomography angiography, and histological examinations support the existence of vascular-structural abnormalities and vascular amyloid β-protein (Aβ) deposits in retinas of AD patients. However, the cellular and molecular mechanisms of such retinal vascular pathology were not previously explored. Here, by modifying a method of enzymatically clearing non-vascular retinal tissue and fluorescent immunolabeling of the isolated blood vessel network, we identified substantial pericyte loss together with significant Aβ deposition in retinal microvasculature and pericytes in AD. Evaluation of postmortem retinas from a cohort of 56 human donors revealed an early and progressive decrease in vascular PDGFRβ in mild cognitive impairment (MCI) and AD compared to cognitively normal controls. Retinal PDGFRβ loss significantly associated with increased retinal vascular Aβ_40_ and Aβ_42_ burden. Decreased vascular LRP-1 and early apoptosis of pericytes in AD retina were also detected. Mapping of PDGFRβ and Aβ_40_ levels in pre-defined retinal subregions indicated that certain geometrical and cellular layers are more susceptible to AD pathology. Further, correlations were identified between retinal vascular abnormalities and cerebral Aβ burden, cerebral amyloid angiopathy (CAA), and clinical status. Overall, the identification of pericyte and PDGFRβ loss accompanying increased vascular amyloidosis in Alzheimer’s retina implies compromised blood–retinal barrier integrity and provides new targets for AD diagnosis and therapy.

**Electronic supplementary material:**

The online version of this article (10.1007/s00401-020-02134-w) contains supplementary material, which is available to authorized users.

## Introduction

Cerebral amyloid angiopathy (CAA) is a complex pathological feature found in over 85% of Alzheimer’s disease (AD) patients involving deposition of amyloid β-protein (Aβ) in blood vessels and other vascular abnormalities [5, 78]. Recent reports implicate cerebral vascular dysfunctions as early and pivotal contributors to the development of AD and CAA as a reliable predictor of cognitive decline [14, 73]. Moreover, studies of brains from AD patients and animal models have described an accelerated degeneration of pericytes [36], vascular cells that regulate blood flow in capillaries [63], and permeability of the blood–brain barrier (BBB), which affected cerebral Aβ accumulation [55, 81]. In addition, brain vascular and perivascular Aβ deposits have also been associated with reduced blood and lymphatic flow [8, 54], impaired gliovascular unit [43], as well as altered vessel diameter and accessibility of peripheral immune cells [49]. These combined processes may lead to reduced Aβ clearance rate, heightened inflammation, and eventually neurodegeneration.

Amyloidosis in cerebral vessel walls predominately consists of Aβ_40_ alloforms [34], which have been implicated in vascular cell toxicity [27]. Along with Aβ_40_, Aβ_42_ alloforms exist in cerebrovascular amyloid deposits of AD patients and in pericytes [34, 55], presumably triggering pericyte loss and thereby affecting these key components of the neurovascular unit. In addition, drainage of Aβ_40_ and Aβ_42_ through the BBB was demonstrated to be one of the primary clearance mechanisms of cerebral Aβ [9]. Removal of Aβ_40_ via the BBB was shown to be mediated by a scavenger receptor LDL receptor-related protein-1 (LRP-1) in mouse models [69]. Importantly, pericyte degeneration as well as LRP-1 downregulation were collectively identified as predominant mechanisms compromising the BBB in AD patients and AD animal models [67, 70]. In fact, pericyte loss, as assessed by pericyte marker platelet-derived growth factor receptor-β (PDGFRβ) in BBB, was tightly associated with functional breakdown of this barrier [61]. This cell surface receptor is also expressed by vascular smooth muscle cells (vSMCs), which are present in all types of blood vessels except for capillaries and pericytic venules [71]. Further, studies in rodents have shown that the loss of PDGFRβ expression alone leads to a decrease in pericyte and vSMC numbers [38] and damaged brain vasculature [61].

Growing evidence shows that AD is not confined within the brain but also affects the retina, a central nervous system (CNS) organ and a developmental outgrowth of the diencephalon [29], which is readily accessible for direct, non-invasive, non-ionizing imaging at high spatial resolution. The neurosensory retina shares many similarities with the brain: both are connected by blood vessels and neuronal axon projections, and contain a large population of neurons, wide-range macro- and micro-glial subtypes, neural fibers, and similar blood barriers comprised of endothelial cells, astrocyte end-feet and pericytes [64]. Evidence from histological examination and noninvasive retinal imaging in living patients with mild cognitive impairment (MCI) and AD reveals that the retina is vastly affected by AD processes. Among key findings were severe optic nerve and retinal ganglion cell (RGC) degeneration, thinning of the retinal nerve fiber layer (RNFL), glial stress, altered electroretinography responses, and vascular abnormalities [26, 32, 37, 47, 76]. Notably, the pathological hallmarks of AD—Aβ plaques and tauopathy—were further identified in the retina of AD patients, including early-stage cases [25, 46, 47, 50]. Noninvasive high-resolution retinal imaging technologies such as fundus imaging, optical coherence tomography (OCT), as well as recently developed OCT angiography [42, 62, 72], retinal amyloid imaging [46–48], and retinal hyperspectral imaging [35, 59] incentivize the use of feasible and inexpensive retinal imaging in the clinical setting to improve AD screening and monitoring.

With regard to retinal vascular changes, a wide range of abnormalities were detected in AD patients [16, 19, 32, 33, 62] including narrowed veins, reduction of blood flow, vascular attenuation, increased width variation, reduction of branching complexity and optimality, reduced arterial fractal dimensions, and increased tortuosity [1, 12, 32]. Moreover, recent studies using OCT and OCT angiography demonstrated that certain retinal vascular abnormalities, both in asymptomatic and clinical AD patients, predicted cognitive impairment [7, 16, 22]. Yet, the cause for these retinal vascular structural changes is still unknown. Interestingly, in histology, it was shown that pathogenic forms of Aβ deposits were often associated with retinal blood vessels and accumulated within and along retinal vasculature [47, 50]. This finding could shed light onto the pathophysiological mechanisms of retinal vascular abnormalities in the AD retina that may involve blood–retina barrier (BRB) disruptions and increased microvascular permeability, possibly leading to neuronal damage. To identify the cellular and molecular components that may be involved in retinal vascular abnormalities in MCI and AD, we conducted an in-depth exploration of retinal vascular amyloidosis and further investigated one of the key components of the BRB—pericytes/PDGFRβ—in relation to cerebral pathology and cognitive status.

Here, we combined fluorescent immunostaining of isolated human retinal vasculature after elastase-based enzymatic digestion of non-vascular tissue to evaluate retinal vascular Aβ deposition and pericyte loss in AD as compared to cognitively normal (CN) controls. We further analyzed a larger cohort (*n* = 56) of postmortem retinal cross-sections and freshly collected retinas from patients with MCI and AD, and compared with age- and sex-matched CN controls. We assessed AD-related pathology in blood vessels across central and peripheral geometrical subregions and layers in pre-defined retinal quadrants. Quantitative analyses were conducted for retinal vascular PDGFRβ expression in pericytes/vSMCs, vascular Aβ_42_ burden, abluminal and vascular Aβ_40_ burden, apoptotic cell markers in pericytes, and retinal LRP-1 expression. Importantly, we compared these retinal parameters with the respective brain pathology and cognitive status. Our findings indicate that along with the substantial increase in retinal vascular amyloidosis in postmortem retinas from AD patients, there was an early and progressive loss of retinal vascular PDGFRβ in pericytes and vSMCs that associated with AD pathology in the brain.

## Materials and methods

### Human eye and brain donors

Donor eyes were obtained from two sources: (1) Alzheimer’s Disease Research Center (ADRC) Neuropathology Core at the Department of Pathology in the University of Southern California (USC, Los Angeles, CA; IRB protocol HS-042071) and (2) National Disease Research Interchange (NDRI, Philadelphia, PA; IRB exempt protocol EX-1055). Both USC-ADRC and NDRI maintain human tissue collection protocols approved by a managerial committee and subject to National Institutes of Health oversight. For a subset of patients and controls we also obtained brain specimens from USC-ADRC. The histological work at Cedars-Sinai Medical Center was performed under IRB protocols Pro00053412 and Pro00019393. Sixty-two postmortem retinas were collected from 29 clinically and neuropathologically confirmed AD patients (age mean ± SD: 81.38 ± 13.79; range 40–98 years; 20 females and 9 males with different disease severities), 11 age- and gender-matched MCI patients (age mean ± SD: 86.45 ± 6.87; range 80–93 years; 5 females and 6 males with different disease severities), and 22 CN individuals (age mean ± SD: 78.18 ± 8.86; range 58–95 years; 13 females and 9 males showing neither clinical cognitive impairment/dementia nor brain pathology). The entire human cohort information is listed in Table 1. The groups had no significant differences in age, sex, or post-mortem interval (PMI) hours. All samples were deidentified and could not be traced back to tissue donors.

**Table 1:** Demographic data for all human eye donors

(*N* = 62)	CN	MCI	AD	*F*	*p*
Subject size	22	11	29	–	–
Females (%), males	13F (59%), 9 M	5F (45%), 6 M	20F (69%), 9 M		
Age ± SD (years)	78.18 ± 8.86	86.45 ± 4.87	81.38 ± 13.79	2.1	0.1
Race (%)	17C (77.3%)	9C (81.8%)	18C (62.1%)	–	–
	1B (4.5%)	1H (9.1%)	1B (3.4%)		
	4 N/A (18.2%)	1B (9.1%)	5A (17.2%)		
			3H (10.3%)		
			2 N/A (6.9%)		
PMI (h)	7.1 ± 2.2	8.9 ± 5.2	7.4 ± 3.7	0.8	0.5

*CN* cognitively normal, *MCI* mild cognitive impairment, *AD* Alzheimer’s disease, *F* female, *M* male, *SD* standard deviation, *C* Caucasian, *B* Black, *H* Hispanic, *A* Asian, *N/A* not available, *PMI* post-mortem interval, Values are presented as mean ± SD. *F* and *p* values were determined by one-way ANOVA with Sidak’s multiple comparison test

### Clinical and neuropathological assessments

The clinical and neuropathological reports provided by the USC ADRC Clinical Core included subjects’ neurological examinations, neuropsychological and cognitive tests, family history, and medication list; psychometric testing was performed by a trained psychometrist under the supervision of a licensed clinical neuropsychologist, following standard-of-care cognitive screening evaluations of patients in their respective neurology clinics, as previously described [21, 47]. NDRI reports provided the medical history of each subject. Most cognitive evaluations were performed annually, and, in most cases, less than 1 year prior to death. Cognitive testing scores from evaluations obtained closest to subjects’ death were used for this analysis. Two global indicators of cognitive status were used for clinical assessment: the Clinical Dementia Rating (CDR; 0 = Normal; 0.5 = Very Mild Dementia; 1 = Mild Dementia; 2 = Moderate Dementia; 3 = Severe Dementia) [60] and the Mini Mental State Examination (MMSE; normal cognitio*n = *24–30, mild dementia = 20–23, moderate dementia = 10–19, severe dementia ≤ 9) [31]. In this study, the clinical diagnostic groups (AD, MCI, and CN) were determined by the source clinicians, based on a comprehensive battery of tests, including neurological examinations, neuropsychological evaluations, and the above-mentioned cognitive tests. For final diagnosis based on the neuropathological reports, the modified Consortium to Establish a Registry for Alzheimer’s Disease [65] was used as outlined in the National Institute on Aging (NIA)/Regan protocols with revision by the NIA and Alzheimer’s Association [39]. Aβ burden (diffuse, immature, or mature plaques), amyloid angiopathy, neuritic plaques, NFTs, neuropil threads, granulovacuolar degeneration, Lewy bodies, Hirano bodies, Pick bodies, balloon cells, neuronal loss, microvascular changes and gliosis pathology were assessed in multiple brain areas: hippocampus (CA1 and CA4), entorhinal cortex, frontal cortex, temporal lobe, parietal lobe, occipital lobe (primary visual cortex, area 17; visual association cortex, area 18), basal ganglia, brainstem (pons, midbrain), cerebellum and substantia nigra.

Amyloid plaques and tangles in the brain were evaluated using anti β-amyloid mAb clone 4G8, Thioflavin-S (ThioS), and Gallyas silver stain in formalin-fixed, paraffin-embedded tissues. Two neuropathologists provided scores based on independent observations of β-amyloid, NFT burden, and/or neuropil threads (0 = none; 1 = sparse 0–5; 3 = moderate 6–20; 5 = abundant/frequent 21–30 or above; N/A = not applicable), and an average of two readings was assigned to each individual. Final diagnosis included AD neuropathologic change (ADNC). Aβ plaque score was modified from Tal et al. (A0 = no Aβ or amyloid plaques; A1 = Thai phase 1 or 2; A2 = Thai phase 3; A3 = Thai phase 4 or 5) [74]. NFT stage was modified from Braak for silver-based histochemistry or p-tau IHC (B0 = No NFTs; B1 = Braak stage I or II; B2 = Braak stage III or IV; B3 = Braak stage V or VI) [15]. Neuritic plaque score was modified from CERAD (C0 = no neuritic plaques; C1 = CERAD score sparse; C2 = CERAD score moderate; C3 = CERAD score frequent) [58]. Neuronal loss, gliosis, granulovacuolar degeneration, Hirano bodies, Lewy bodies, Pick bodies, and balloon cells were evaluated (0 = absent; 1 = present) in multiple brain areas using hematoxylin and eosin (H&E) staining. Amyloid angiopathy was graded as follows: Grade I = amyloid restricted to a rim around normal/atrophic SMCs of vessels; Grade II = media replaced by amyloid and thicker than normal, but no evidence of blood leakage; Grade III = extensive amyloid deposition with focal vessel wall fragmentation and at least one focus of perivascular leakage; Grade IV = extensive amyloid deposition and fibrinoid necrosis, micro aneurysms, mural thrombi, lumen inflammation, and perivascular neuritis. For the correlation analyses against retinal parameters, we used the following CAA scoring system: no amyloid angiopathy was assigned ‘0’; grade I was assigned as ‘1’, grade I–II as ‘1.5’, grade II as ‘2’, and grade II–III as ‘2.5’.

### Collection and processing of eyes and cortical tissues

Donor eyes were collected within 7 h, on average, from time of death and were either preserved in Optisol-GS media (Bausch & Lomb, 50,006-OPT) and stored at 4 °C for less than 24 h, fresh frozen (snap; stored at − 80 °C), or punctured once and fixed in 10% neutral buffered formalin (NBF) or 2.5% Paraformaldehyde (PFA) and stored at 4 °C. Brain tissues (hippocampus; occipital lobe – primary visual cortex, area-17, and frontal cortex, area-9) from the same donors were snap frozen and stored at − 80 °C. Parts from the fresh-frozen brain tissues were fixed in 4% PFA for 16 h following dehydration in 30% sucrose/PBS. Brain tissues were cryosectioned (30 μm thick) and placed in phosphate-buffered saline 1x (PBS) with 0.01% sodium azide (Sigma-Aldrich) at 4 °C. Irrespective of the human donor eye source, USC-ADRC or NDRI, the same tissue collection and processing methods were applied.

### Preparation of retinal flatmounts and strips

Fresh-frozen eyes and eyes preserved in Optisol-GS were dissected with anterior chambers removed to create eyecups. Vitreous humor was thoroughly removed manually. Retinas were dissected out, detached from the choroid, and flatmounts were prepared [47]. By identifying the macula, optic disc, and blood vessels, the geometrical regions of the four retinal quadrants were defined with regard to the left and the right eye. Flatmount strips (2–3 mm in width) were dissected along the retinal quadrant margins to create four strips: superior-temporal—ST, inferior-temporal—TI; inferior-nasal—IN, and superior-nasal—NS, and were fixed in 2.5% PFA for cross-sectioning. In a subset of human eye donors, a second set of strips was prepared (5 mm in width) and stored at − 80 °C for protein analysis. Each strip was approximately 2–2.5 cm long from the optic disc to the ora serrata and included the central, mid, and far retinal areas. All the above stages were performed in cold PBS with 1 × Protease Inhibitor cocktail set I (Calbiochem 539,131). Eyes that were initially fixed in 10% NBF or 2.5% PFA were dissected to create eyecups, and the retinas were dissected free. Vitreous humor was thoroughly removed and flatmounts were prepared. As described above, a set of flatmount strips (ST, TI, IN, and NS) was dissected (2–3 mm in width), washed in PBS, and processed for retinal cross-sectioning.

### Retinal cross-sections

Flatmount strips were initially embedded in paraffin using standard techniques, then rotated 90° horizontally and embedded in paraffin. The retinal strips were sectioned (7–10 µm thick) and placed on microscope slides that were treated with 3-Aminopropyltriethoxysilane (APES, Sigma A3648). Before immunohistochemistry, the sections were deparaffinized with 100% xylene twice (for 10 min each), rehydrated with decreasing concentrations of ethanol (100–70%), and then washed with distilled water followed by PBS.

### Retinal vascular isolation and immunofluorescent staining

**Fig. 1:**
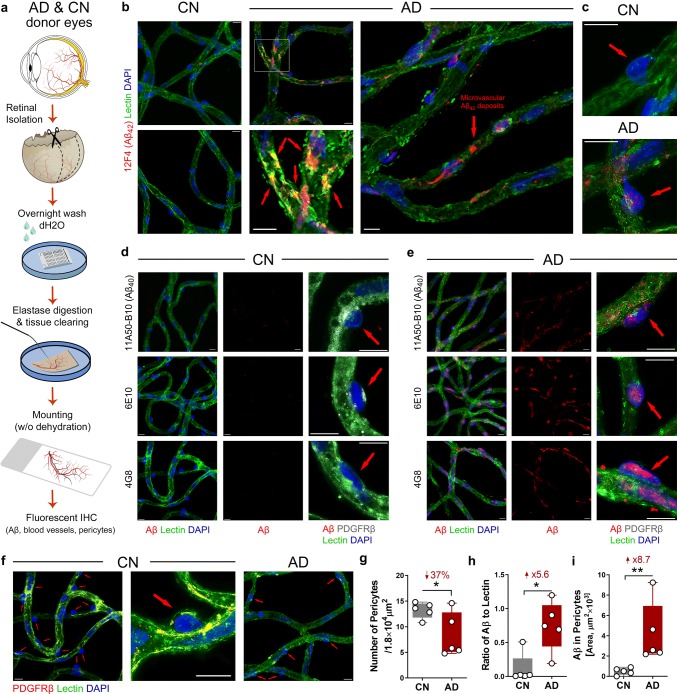
Microvascular network from postmortem retinas of AD patients exhibit pericyte loss along with Aβ accumulation in blood vessels and pericytes. **a** Schema of modified retinal vascular network isolation and immunofluorescent staining. Whole retinas were isolated from donor eyes and 7 mm wide strips were prepared from the temporal retinal hemisphere spanning from the ora serrata to the optic disk. Following fixation, washing, and elastase digestion, vascular network is mounted onto slides without dehydration. Immunofluorescent staining was applied on isolated retinal vascular network to detect Aβ (6E10, 4G8, 12F4 and 11A50), pericytes (PDGFRβ), and blood vessels (lectin). **b**, **c** Representative fluorescent images of isolated retinal microvasculature stained for Aβ_42_ (12F4, red), blood vessels (lectin, green), and nuclei (DAPI, blue) in age- and sex-matched human donors with AD (*n* = 5) and cognitively normal (CN, *n* = 5). Arrows indicate microvascular Aβ_42_ deposits in capillaries on panel **b** [a zoomed-in image of AD donor retina (lower image) shows co-localization of Aβ_42_ and retinal vascular wall; yellow spot], or pericytes on panel **c**; **d**, **e** Representative fluorescent images of isolated retinal microvasculature stained for Aβ (11A50-B10, 6E10 or 4G8, red), pericytes (PDGFRβ, white), blood vessels (lectin, green), and nuclei (DAPI, blue) in age- and sex-matched AD and CN human donors. Arrows indicate pericytes. **f** Representative fluorescent images of isolated retinal microvasculature stained for pericytes (PDGFRβ, red), blood vessels (lectin, green), and nuclei (DAPI, blue). Arrows indicate pericytes. **g**–**i** Quantitative analyses of **g** mean number of retinal pericytes in each microscopic visual field (1.8 × 10^4^ µm^2^ area), **h** ratio of retinal vascular Aβ immunoreactive (IR) area to lectin IR area from each microscopic visual field (1.8 × 10^4^ µm^2^ area), and **i** Aβ IR area within pericytes, in the same cohort of AD (*n* = 5) and CN (*n* = 5) human donors. Scale bars = 10 μm. Data from individual subjects as well as group mean ± SEM are shown. Fold and percent changes are shown in red. *p < 0.05, **p < 0.01, determined by unpaired two tailed Student’s *t* test

We modified the retinal vascular isolation method to use on human retinal tissues and immuno-fluorescently label pericytes and amyloidosis (illustrated in Fig. 1a). This trypsin-induced retinal digestion and vascular network isolation technique was originally developed in 1993 [51] and subsequently modified by replacing trypsin with commercially available elastase [77]. Our modified protocol is as follows: retinal strips from human donors or mouse whole retinas preserved in PFA were first washed in lukewarm running distilled water overnight, then digested in 40 U/ml elastase solution (Merck Millipore, Burlington, MA) for 2 h at 37 °C. After digestion, tissues were incubated in activation solution (Tris buffer at pH 8.5) overnight for extensive digestion. The next day, retinas were transferred to superfrost microscope slides with 1 × PBS, then carefully cleaned with rat whisker to remove unwanted tissues under a dissecting microscope. After cleaning non-vascular tissues, 1 × PBS was applied three times to wash the isolated vascular tissues. When we were able to observe clean vascular tree on slides under dissecting microscope, tissues were mounted on slides carefully without dehydration, then incubated in blocking buffer (Dako #X0909) for 1 h at room temperature (RT). Primary antibodies were applied to the tissue after blocking, then incubated at 4 °C overnight as listed (antibody information provided in Table 2): 4G8/lectin/PDGFRβ, 6E10/lectin/PDGFRβ, 11A50-B10/lectin/PDGFRβ, 12F4/lectin/PDGFRβ. Tissues were then washed three times by PBS and incubated with secondary antibodies against each species (information provided in Table 2) for 2 h at RT. After rinsing with PBS three times, vascular trees were mounted by Prolog Gold antifade reagent with DAPI (Invitrogen #P36935). For quantification purposes, images were taken on a Carl Zeiss Axio Imager Z1 fluorescence microscope (Carl Zeiss MicroImaging, Inc.) equipped with ApoTome, AxioCam MRm, and AxioCam HRc cameras (for more details see “Stereological quantification” below). For representative images, Z-stack images were repeatedly captured at same tissue thickness using a Carl Zeiss 780 Confocal microscope (Carl Zeiss MicroImaging, Inc.). Routine controls were processed using identical protocols while omitting the primary antibody to assess nonspecific labeling. Representative images of all negative controls are shown in Supplementary Fig. 1, online resource.

**Table 2:** List of antibodies used in the study

Antigen and clone	Source species	Dilution	Commercial source	Catalog. #
*Primary antibody*				
PDGFRβ pAb	Goat	1:200	R&D Systems	AF385
Aβ_40_ (11A50-B10) mAb	Mouse	1:250 DAB	Biolegend	805401
		1:200 FL		
JRF/cAβ_40/28_ # 8152 mAb	Mouse	1:2500 DAB	Janssen Pharmaceutica	N/A
		1:2000 FL		
CD31 pAb	Rabbit	1:50	Abcam	ab28364
Aβ_42_ (12F4) mAb	Mouse	1:200	Biolegend	805501
4G8 mAb	Mouse	1:200	Biolegend	800701
6E10 mAb	Mouse	1:200	Biolegend	803001
Caspase-3 pAb	Rabbit	1:500	Abcam	ab13847
Cleaved Caspase-3 pAb	Rabbit	1:200	Cell Signaling	9661
LRP-1 mAb	Rabbit	1:200	Abcam	ab92544
Alexa Fluor 488-conjugated tomato lectin	Lycopersicon esculentum	1:200	Dylight	DL-1174
*Secondary antibody*				
Cy3 (anti-rabbit, anti-goat)	Donkey	1:200	Jackson ImmunoResearch Laboratories	
Cy5 (anti-mouse, anti-rabbit)	Donkey	1:200	Jackson ImmunoResearch Laboratories	
Alexa 647 (anti-goat)	Donkey	1:200	Jackson ImmunoResearch Laboratories	

*IHC* immunohistochemistry, *ICC* immunocytochemistry, *pAb* polyclonal antibody, *mAb* monoclonal antibody, *DAB* peroxidase-based immunohistochemistry visualized with DAB substrate; *FL* fluorescence-based immunohistochemistry. If not marked otherwise, antibody dilution is indicated for immunofluorescent essay

### Mice

The double-transgenic B6.Cg-Tg (APP_SWE_/PS1_∆E9_)85Dbo/Mmjax hemizygous (ADTg) mice strain (MMRRC stock #34832-JAX|APP/PS1) and their non-Tg littermates (as WT control non-AD) were used for retinal vascular isolation experiments. All mice are on the genetic background of B6. Mice were purchased from MMRRC and later bred and maintained at Cedars-Sinai Medical Center. The mouse experiments were conducted in accordance with Cedars-Sinai Medical Center Institutional Animal Care and Use Committee (IACUC) guidelines under an approved protocol. We used a total of nine 8.5-month-old mice (all males) divided into three groups: perfused WT (*n* = 3), perfused ADTg (*n* = 3), and non-perfused ADTg (*n* = 3) mice. Animals were deeply anesthetized under Ketamine/Xylazine (40–50 mg/kg) before being euthanized either by transcardial perfusion (0.9% ice-cold sodium chloride supplemented with 0.5 mM EDTA) or cervical dislocation (non-perfused group). Eyes were dissected and the retinas were immediately isolated. Using a 25-gauge needle, a hole is poked in the cornea and an incision is made along the ora serrata to remove the lens and cornea-iris. Next, a small incision is made in the sclera-choroid layers toward the optic nerve and using fine forceps, sclera and choroid is gently separated from the retina, which is cleanly snipped at its base from the optic nerve. Care is taken to isolate whole retina undamaged to preserve vasculature network. Following isolation, retinas were fixed in 4% PFA for 7 days. Retinas were then processed for retinal vascular isolation and immunofluorescent staining as described above.

### Biochemical determination of Aβ_1–40_ levels by sandwich ELISA

Frozen human retinal flatmount strips from the temporal hemisphere (ST, TI) were weighed and placed in a tube with cold homogenization buffer [Tris/EDTA buffer pH 9 (DAKO, S2368), 1% Triton X-100 (Sigma, T8787), 0.1% NaN_3_ (Sigma, 438456) and 1 × Protease Inhibitor cocktail set I (Calbiochem 539131)], then homogenized by sonication (Qsonica Sonicator M-Tip, Amplitude 4, 6 W, for 90 s; sonication pulse was stopped every 15 s to allow the cell suspension to cool down for 10 s). The ultrasonic probe positioned inside the tube was placed in ice water. Next, retinal strip homogenates were incubated for 1 h at 98 °C in a water bath. After determination of the protein concentration (Thermo Fisher Scientific), retinal Aβ_1–40_ was determined using an anti-human Aβ_1–40_ end-specific sandwich ELISA kit (Thermo Fisher, KHB3481).

### Immunofluorescent staining of retinal cross-sections

After deparaffinization, retinal cross-sections were treated with antigen retrieval solution at 98 °C for 1 h (PH 6.1; Dako #S1699) and washed in PBS. Retinal sections were then incubated in blocking buffer (Dako #X0909), followed by primary antibody incubation (information provided in Table 2) overnight in 4 °C with the following combinations: PDGFRβ (1:200)/lectin (1:200)/11A50-B10 (1:200), PDGFRβ (1:200)/lectin (1:200)/12F4 (1:200), CD31 (1:50)/JRF/cAβ 40/28 #8152 (1:2000), LRP-1 (1:200)/PDGFRβ (1:200)/lectin (1:200), cleaved caspase-3 (1:200)/PDGFRβ (1:200)/lectin (1:200). Alexa Fluor 488-conjugated tomato lectin was used to visualize blood vessel cells. Retinal sections were then washed three times by PBS and incubated with secondary antibodies against each species (1:200, information provided in Table 2) for 2 h at RT. After rinsing with PBS for three times, sections were mounted with Prolong Gold antifade reagent with DAPI (Thermo Fisher #P36935). Images were repeatedly captured at the same focal planes with the same exposure time using a Carl Zeiss Axio Imager Z1 fluorescence microscope (Carl Zeiss MicroImaging, Inc.) equipped with ApoTome, AxioCam MRm, and AxioCam HRc cameras. Images were captured at 20 ×, 40 ×, and 63 × objectives for different purposes (for more details see “Stereological quantification” below). Routine controls were processed using identical protocols while omitting the primary antibody to assess nonspecific labeling. Representative images of negative controls are shown in Supplementary Fig. 1, online resource.

### Peroxidase-based immunostaining of Aβ

Fixed brain sections and retinal cross-sections after deparaffinization were treated with target retrieval solution (pH 6.1; S1699, DAKO) at 98 °C for 1 h and washed with PBS. In addition, treatment with 70% formic acid (ACROS) for 10 min at RT was performed on brain sections and retinal cross-sections before staining for Aβ. Peroxidase-based immunostaining was performed. For antibodies’ list and dilutions, see Table 2. Prior to peroxidase-based immunostaining, the tissues were treated with 3% H_2_O_2_ for 10 min, and two staining protocols were used: (1) Vectastain Elite ABC HRP kit (Vector, PK-6102, Peroxidase Mouse IgG) according to manufacturer’s instructions or (2) All Dako reagents protocol. Following the treatment with formic acid, the tissues were washed with wash buffer (Dako S3006) for 1 h, then treated with H_2_O_2_ and rinsed with wash buffer. Primary antibody (Ab) was diluted with background reducing components (Dako S3022) and incubated with the tissues for 1 h at 37 °C for JRF/cAβ 40/28 # 8152, or overnight at 4 °C for 11A50–B10 (Aβ_40_) mAbs. Tissues were rinsed twice with wash buffer on a shaker and incubated for 30 min at 37 °C with secondary Ab (goat anti mouse ab HRP conjugated, DAKO Envision K4000), then were rinsed again with wash buffer. For both protocols, diaminobenzidine (DAB) substrate was used (DAKO K3468). Counterstaining with hematoxylin was performed followed by mounting with Faramount aqueous mounting medium (Dako, S3025). Routine controls were processed using identical protocols while omitting the primary antibodies to assess nonspecific labeling. Representative images of negative controls are shown in Supplementary Fig. 1, online resource.

### Transmission electron microscopy (TEM) analysis

Analyses of a retinal whole mount from an AD donor retina that was pre-stained with anti-Aβ_42_ mAb (12F4) and a high-sensitivity immunoperoxidase-based system with 3,3′ Diaminobenzidine (DAB) substrate chromogen were performed using transmission electron microscopy. Stained tissues were processed for electron microscopic imaging; the samples were dehydrated in serially graded ethanol and then infiltrated in Eponate 12 (Ted Pella, Inc. Redding, CA, USA) prior to embedding between two acetate sheets. Ultrathin sections of retina were cut into cross sections at a thickness of 70 nm, examined on a JEOL JEM 2100 (JEOL USA, Peabody, MA, USA), and photographed with the Orius SC1000B digital camera (Gatan, Pleasanton, CA, USA). Images were processed and colorized using Adobe Photoshop CS4 (Adobe Inc., San Jose, CA, USA).

### TUNEL assay for detection of apoptotic retinal pericytes

Formalin-fixed paraffin-embedded retinal cross-sections after deparaffinization were washed with PBS and then incubated with Proteinase-K (Recombinant PCR grade, 15 µg/ml in 10 mM Tris/HCL pH 7.6; Roche Diagnostics GmbH; 03115836001) at 37 °C for 20 min. Next, slides were washed with PBS and incubated with TUNEL reaction mixture (50 µl on each slide; Roche Diagnostics GmbH; 11684795910) at 37 °C for 60 min, in a humidified chamber in dark (the samples were covered with parafilm to ensure a homogeneous spread of TUNEL reaction and to avoid evaporation loss). Afterward, slides were washed with PBS and fluorescent-based immunostaining was performed using blocking solution (DAKO X0909) for 45 min at RT. The tissues were incubated with primary antibody, goat anti PDGFRβ, overnight at 4 °C, then the secondary antibody, donkey anti goat Alexa 647, was applied for 1 h at RT. Then, the samples were washed with PBS and covered with ProLong™ Gold antifade mounting media with DAPI (Molecular Probes; #P36935). Negative and positive controls were included (see Supplementary Fig. 1, online resource) in this experimental setup: for TUNEL negative control the retinal tissues were incubated with only 50 µl of TUNEL label solution (without the TUNEL Enzyme solution-terminal transferase) instead of TUNEL reaction mixture. For TUNEL positive control the retinal tissues were incubated with DNase I (1000 U/ml in 50 mM Tris–HCL, pH 7.5; Worthington Biochemical Corp. Code D) to induce DNA strand breaks, prior to labeling procedure. The retinal tissue sections were then evaluated under fluorescent microscope.

### Stereological quantification

For Fig. 1 of isolated retinal blood vessels, quantification was performed from 5 AD donors and 5 age- and sex-matched CN controls. The fluorescence of specific signals was captured using the same setting and exposure time for each image and human donor, with a Z-stack of 10 µm thickness using Axio Imager Z1 microscope (with motorized Z-drive) with AxioCam MRm monochrome camera ver. 3.0 (at a resolution of 1388 × 1040 pixels, 6.45 µm × 6.45 µm pixel size, dynamic range of > 1:2200 that delivers low-noise images due to Peltier-cooled sensor). Images were captured at 40 × objective, at respective resolution of 0.25 µm. Fifteen images were taken randomly from each region of central, mid-, and far-peripheral retina (five from each region) per subject. Acquired images were converted to grayscale and standardized to baseline using a histogram-based threshold in the NIH ImageJ software (version 1.52o). For each biomarker, total area of immunoreactivity was determined using the same threshold percentage from the baseline in ImageJ (with same percentage threshold setting for all diagnostic groups). The images were then subjected to particle analysis for lectin and Aβ to determine IR area. Pericyte number was based on 15 images, averaging the number in each microscopic visual field (covering 1.8 × 10^4^ µm^2^ area), per human donor. We used the grid mode in ImageJ to manually count the number of pericytes. The ratio of Aβ to lectin was calculated by dividing Aβ IR area by lectin IR area in each of the 15 images (described above) and averaging the values per human donor. The sum of Aβ IR area from an identical number of randomly selected pericytes (*n* = 10) from each human donor was used to calculate Aβ in pericytes. An identical region of interest was used for the standardized histogram-based threshold technique and subjected to particle analysis.

**Fig. 2:**
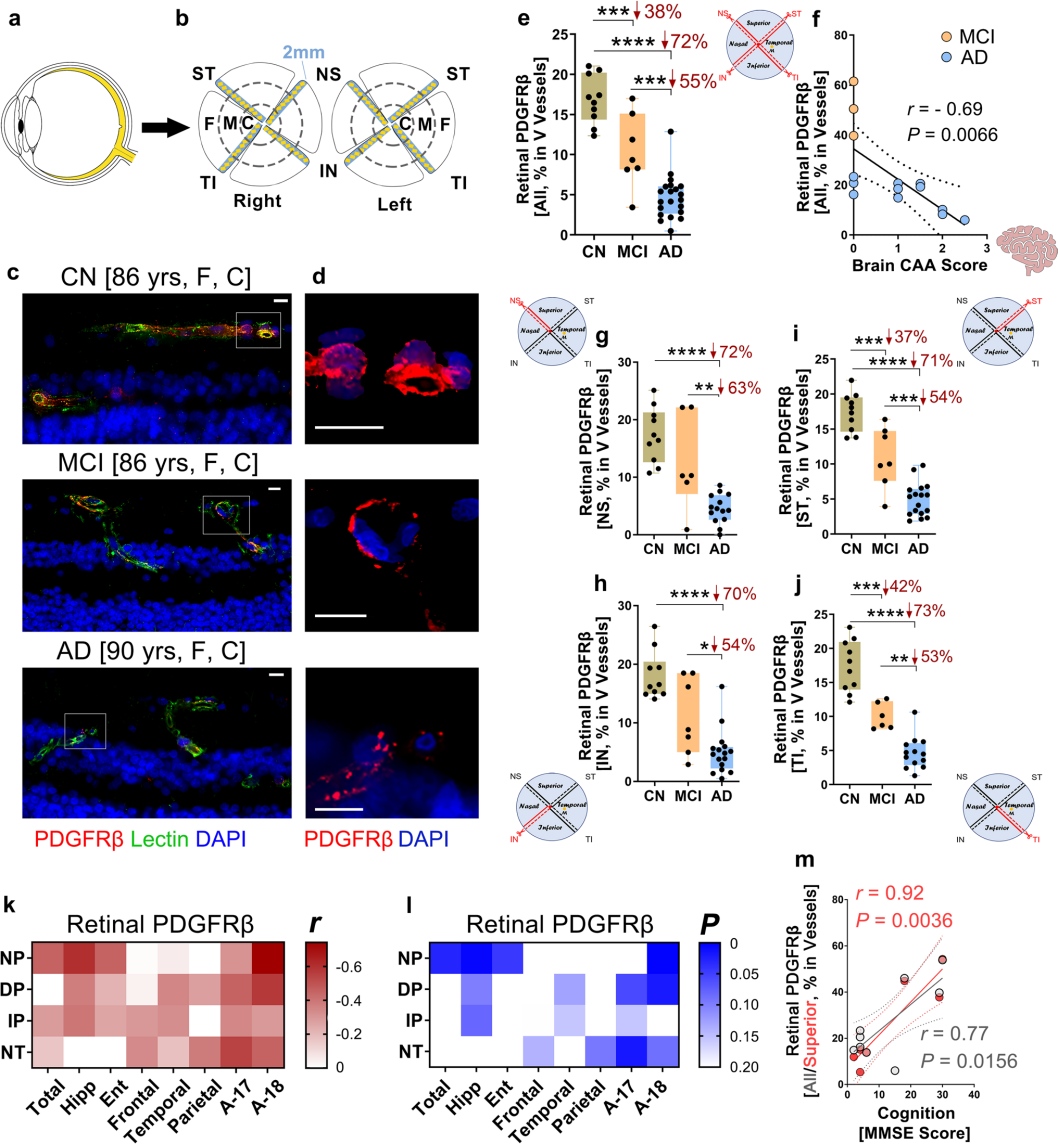
Early and progressive loss of retinal vascular PDGFRβ in MCI and AD. **a**, **b** Schematic diagram of donor eye dissection, isolation of neurosensory retina (yellow), and retinal processing for histological analysis. Anatomically defined strips from all four quadrants, superior-temporal—ST, temporal-inferior—TI, inferior-nasal—IN, and nasal-superior—NS, were prepared and analyzed in pre-determined geometrical regions: central (C), mid- (M) and far- (F) periphery. **c**, **d** Representative fluorescent images of paraffin-embedded retinal cross-sections stained for PDGFRβ (red), with blood vessels (lectin, green) and nuclei (DAPI, blue) in age- and sex-matched human donors with AD, mild cognitive impairment (MCI), and cognitively normal (CN; *yrs *years, *F* female, *C* Caucasian). **c** Longitudinal (L) blood vessels (~ 10 µm in diameter); **d** Zoomed-in PDGFRβ^+^ vascular cells are shown from selected regions (dashed white rectangle in **c**). Scale bars = 10 μm. **e** Quantitative analysis of percent PDGFRβ IR area in vertical (V) blood vessels in the retinas of donors with AD (*n* = 21), MCI (*n* = 7), and CN (*n* = 10). **f** Pearson’s coefficient (*r*) correlation between percent retinal PDGFRβ IR area in sum of V and L blood vessels against CAA scores in a subset of AD (*n* = 11) and MCI (*n* = 3) human donors. **g–j** Quantitative analysis of percent PDGFRβ immunoreactive (IR) area in V vessels from each retinal quadrant separately: **g** NS, **h** IN, **i** ST, **j** TI, in the same human cohort as in (**e**). **k–l** Heat-map illustrating Pearson’s correlations between percent retinal PDGFRβ IR area and brain pathology, including neuritic plaques (NP), diffuse plaques (DP), immature plaques (IP), and neuropil threads (NT), in AD (*n* = 14), MCI (*n* = 5) and CN (*n* = 1) human subjects (*n* = 20 total). Pseudo-color **k** red for (*r*) values and **l** blue for (*P*) values demonstrate the strength of each correlation parameter; Total—all brain regions averaged, Hipp—hippocampus, Ent—entorhinal cortex, Frontal—frontal cortex, Temporal—temporal cortex, Parietal—parietal cortex, A-17—primary visual cortex, and A-18—visual association cortex. **m** Correlation between percent retinal PDGFRβ IR area of all (mean of four quadrants; gray dots) or superior retinal hemisphere (mean of ST and NS; red dots) against the mini-mental state examination (MMSE) cognitive scores (*n* = 10). Data from individual subjects as well as group mean ± SEM are shown. Percent changes are shown in red. *p < 0.05, **p < 0.01, ***p < 0.001, ****p < 0.0001, by one-way ANOVA with Sidak’s post-hoc multiple comparison test

**Fig. 3:**
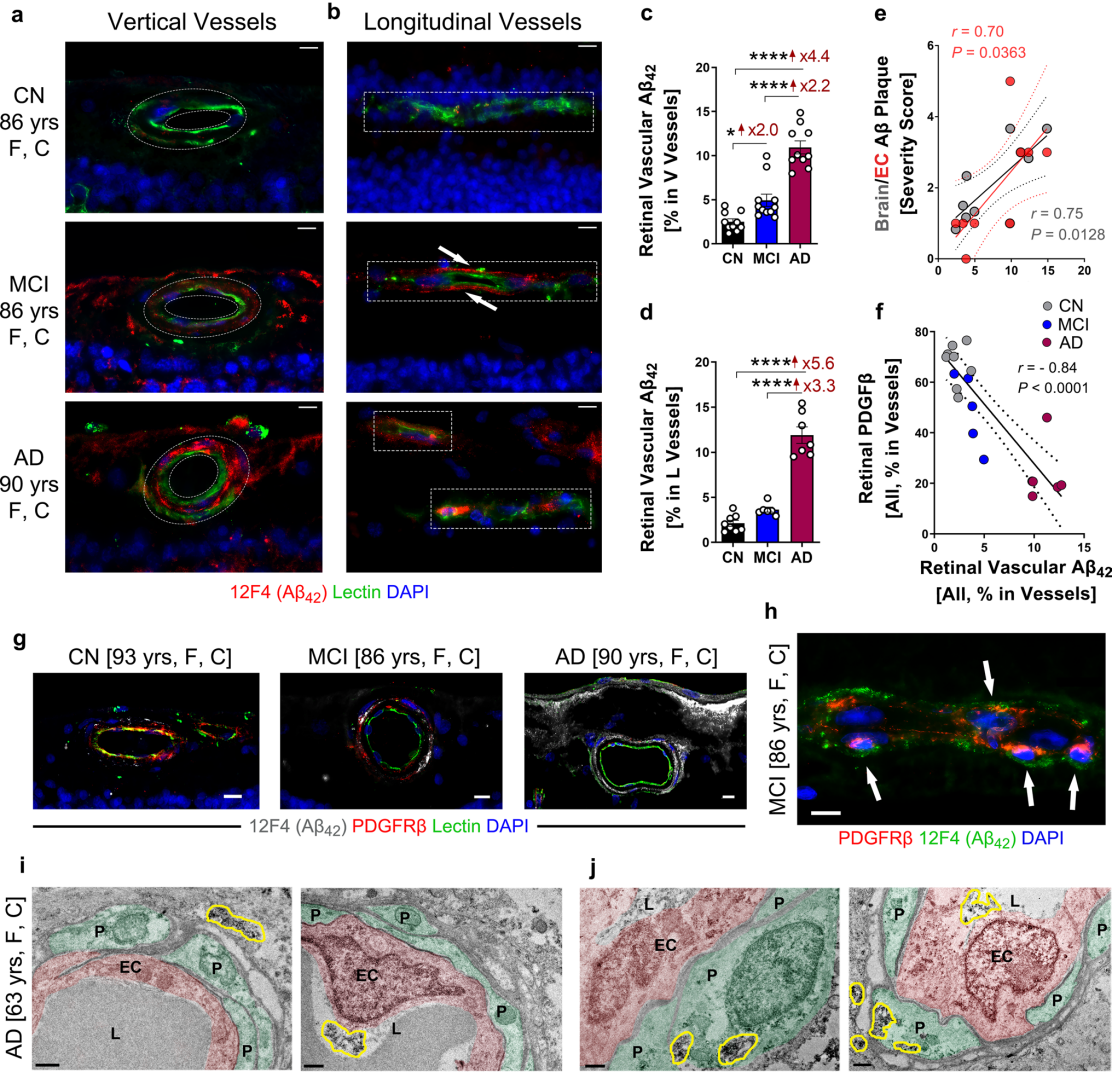
Increased vascular Aβ_42_ including in pericytes is tightly associated with PDGFRβ loss in postmortem retinas of MCI and AD patients. **a**, **b** Representative fluorescent images of paraffin-embedded retinal cross-sections isolated from human donors with AD, MCI, or cognitively normal (CN) stained for Aβ_42_ (12F4, red), blood vessels (lectin, green), and nuclei (DAPI, blue). **a** Vertical (V) and **b** longitudinal (L) vessels are shown (*yrs* years; *F* female; *C* Caucasian); geometric shapes in white dashed lines indicate pre-defined areas of analysis. Scale bars = 10 μm. **c**, **d** Quantitative analysis of percent 12F4 immunoreactive (IR) area in retinal **c** V or **d** L blood vessels in age- and sex-matched human donors with AD (*n* = 10), MCI (*n* = 11) and CN (*n* = 10). **e**, **f** Pearson’s coefficient (*r*) correlation between retinal 12F4^+^Aβ_42_ burden in average of V and L blood vessels against **e** neuritic Aβ plaques either in whole brain (gray dots) or entorhinal cortex (EC; red dots) and **f** percent retinal vascular PDGFRβ IR area within a subset of human donors with AD, MCI and CN (*n* = 8, *n* = 10, and *n* = 18, respectively). **g** Representative fluorescent images of retinal vertical vessels from human eye donors with AD, MCI, or CN, stained for Aβ_42_ (12F4, white), PDGFRβ (red), blood vessels (lectin, green), and DAPI for nuclei (blue). Scale bars = 10 μm. **h** A microscopic image of longitudinal vessel from MCI retina showing vascular Aβ_42_ immunoreactivity (green) co-localized with PDGFRβ^+^ cells (red, arrows). Scale bars = 10 μm. **i**, **j** Transmission electron microscopy (TEM) images of retinal vertical-sections from an AD human donor; retina was pre-stained with anti-Aβ_42_ mAb (12F4) and an immunoperoxidase-based DAB. TEM analysis reveals the location and ultrastructure of retinal vascular-associated Aβ deposits (demarcated by yellow shapes). **i** Left, retinal Aβ_42_ deposit in the outer vascular surface adjacent to pericytes (P, green), with a clean blood vessel lumen (L). Right, retinal Aβ_42_ deposited inside a blood vessel lumen attached to an endothelial cell (EC, pink) surface. **j** Retinal Aβ_42_ deposits within pericytes, detected in the cytoplasm and adjacent to mitochondria, as well as on vessel outer surface external to the pericytes. Scale bars = 0.5 µm. Data from individual human donors as well as group mean ± SEM are shown. Fold changes are shown in red. *p < 0.05, **p < 0.01, ***p < 0.001, ****p < 0.0001, by one-way ANOVA with Sidak’s post-hoc multiple comparison test

**Fig. 4:**
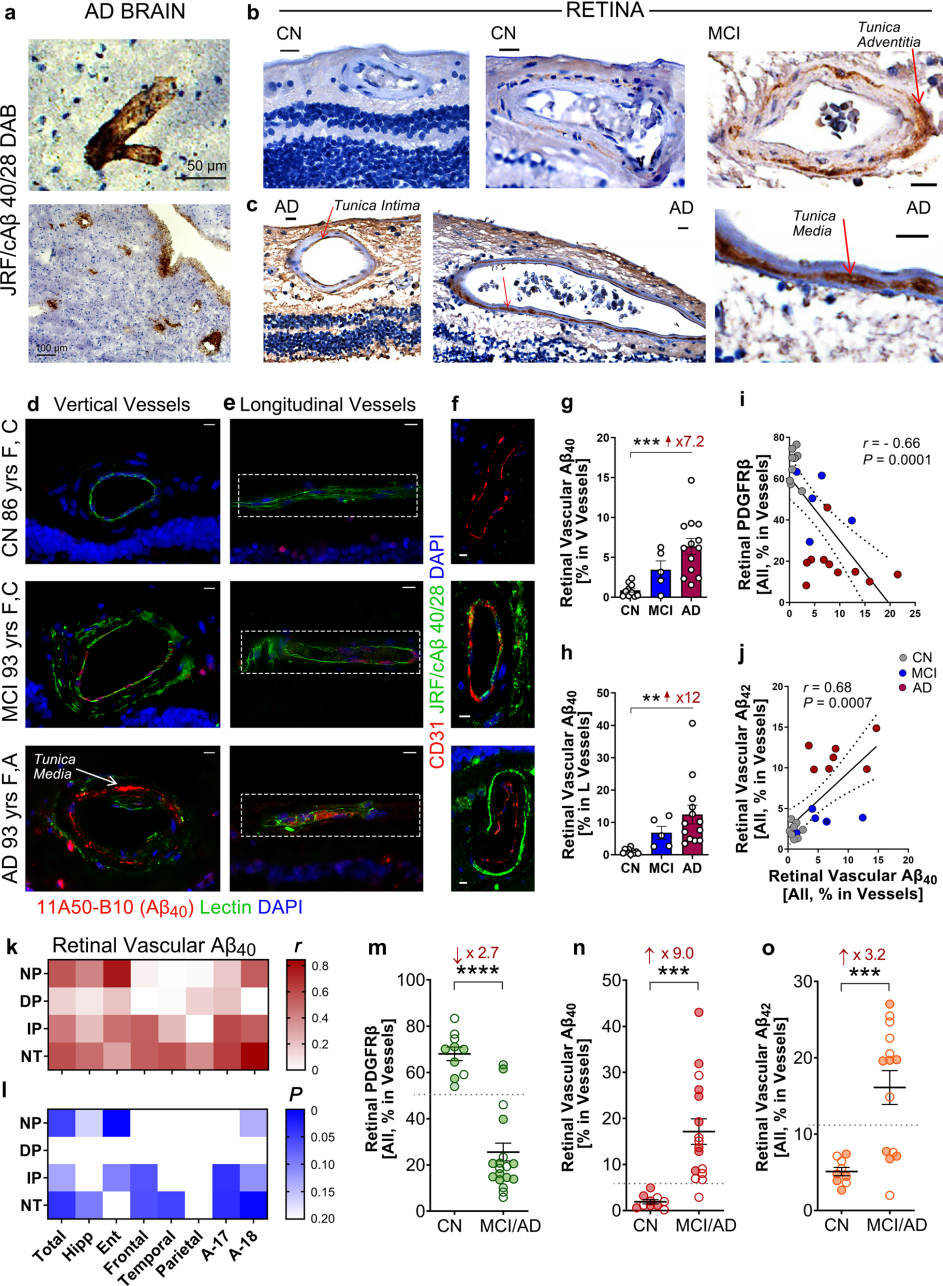
Retinal vascular Aβ_40_ burden in AD retina correlates with both retinal vascular Aβ_42_ deposits and PDGFRβ loss and can predict disease status. **a–c** Representative images of retinal and brain sections immunostained against Aβ_40_ (JRF/cAβ_40/28_; #8152) with DAB labeling and hematoxylin counterstain in cohorts of AD, MCI, and cognitively normal (CN) controls. **c** Arrows indicate vascular Aβ_40_ staining in tunica media, adventitia, or intima; right image is an enlargement of area indicated by arrow from the middle image. Scale bars = 20 µm. **d**, **e** Representative fluorescent images of paraffin-embedded retinal cross-sections isolated from human donors with AD, MCI, or CN (*yrs* years old, *F* female, *C* Caucasian, *A* Asian) and stained for Aβ_40_ (11A50–B10, red), blood vessels (lectin, green), and nuclei (DAPI, blue) in **d** vertical (V) and **e** longitudinal (L) retinal blood vessels. Dashed geometric white shapes indicate pre-defined areas of analysis. Scale bars = 10 µm. **f** Representative microscopic images showing V vessels labeled against endothelial cells (CD31, red), Aβ_40_ (JRF/cAβ_40/28_, green), and nuclei (DAPI, blue) in retinas from AD, MCI, and CN human donors. Scale bars = 10 µm. **g**, **h** Quantitative analysis of percent 11A50-B10^+^Aβ_40_ immunoreactive (IR) area in retinal **g** V and **h** L blood vessels from AD (*n* = 13), MCI (*n* = 5) and CN controls (*n* = 10). **i**, **j**. Pearson’s coefficient (*r*) correlation between retinal Aβ_40_ burden (mean of both V and L vessels) against **i** percent retinal PDGFRβ IR area (*n* = 24 human donors) or **j** percent retinal vascular 12F4^+^Aβ_42_ burden (*n* = 20 human donors). **k**, **l** Heat-map illustrating correlations between percent retinal vascular Aβ_40_ IR area (average of V and L blood vessels) against brain pathology, including neuritic plaques (NP), diffuse plaques (DP), immature plaques (IP), and neuropil threads (NT), in AD (*n = *8), MCI (*n = *3), and CN (*n = *1) human donors (*n = *12 total). Pseudo-color **k** red (*r*) values and **l** blue (*P*) values demonstrate the strength of each correlation parameter; total—average of all brain regions, Hipp—hippocampus, Ent—entorhinal cortex, Frontal—frontal cortex, Temporal—temporal cortex, Parietal—parietal cortex, A-17—primary visual cortex, and A-18—visual association cortex. **m–o** Analysis of retinal parameters when samples are stratified per two diagnostic groups, MCI/AD and CN. **m** Retinal vascular PDGFRβ (*n = *20 MCI/AD and *n = *10 CN). **n** Retinal vascular Aβ_40_ (*n = *16 MCI/AD and *n = *10 CN). **o** Retinal vascular Aβ_42_ (*n = *14 MCI/AD and *n = *9 CN). Dotted lines display the suggested values to separate between control and disease groups. Males in filled circles and Females in clear circles. Data from individual human subjects as well as group mean ± SEM are shown. Fold and percent changes are shown in red. **p < 0.01, ***p < 0.001, ****p < 0.0001, by one-way ANOVA with Sidak’s post-hoc multiple comparison test

**Fig. 5:**
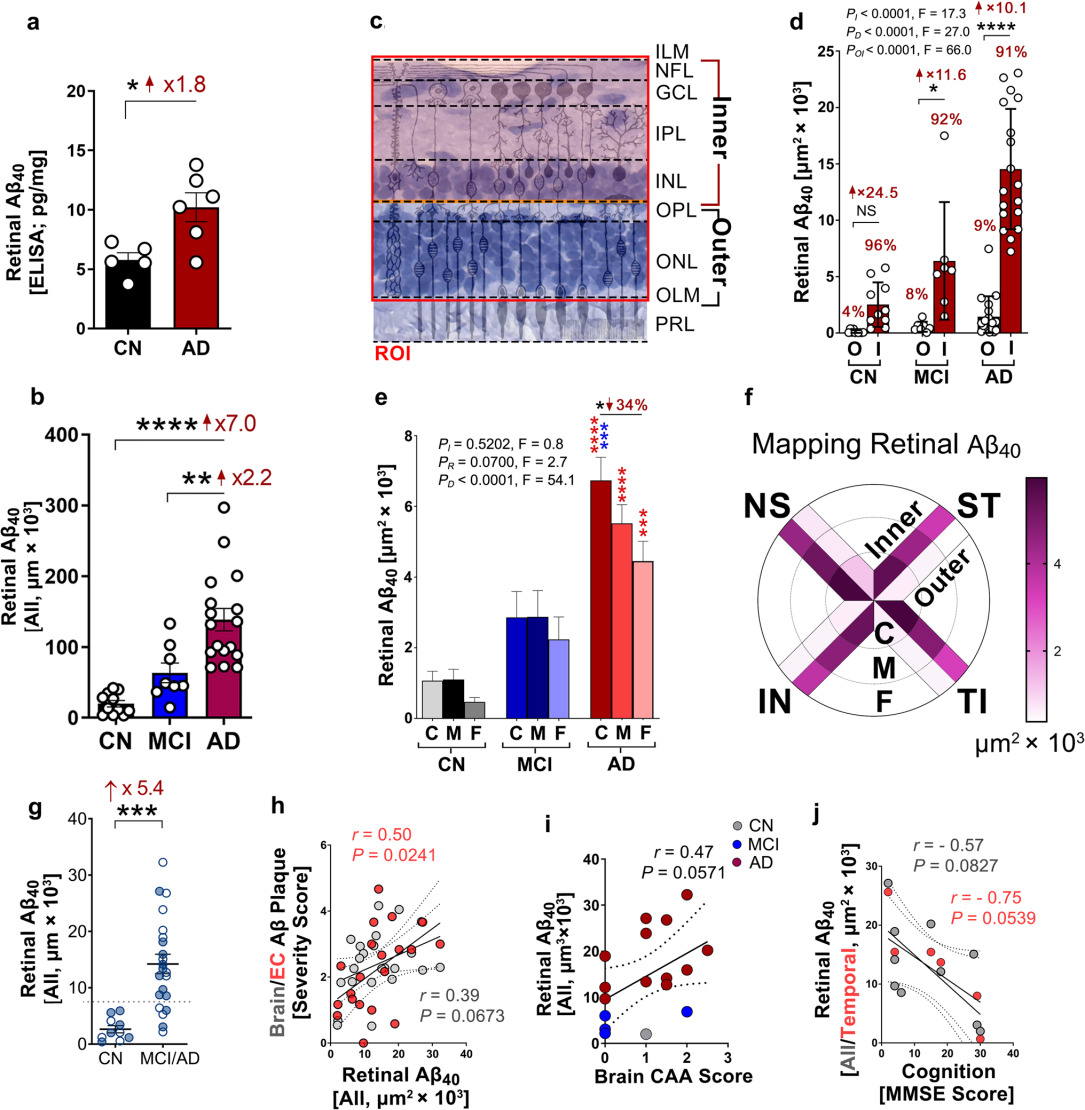
Mapping of retinal Aβ_40_ burden and distribution in predefined geometrical regions and layers. **a** Retinal Aβ_1–40_ concentrations determined by ELISA assay in protein homogenates from postmortem retinas freshly collected from AD patients (*n = *6) and cognitively normal controls (CN, *n = *5). **b** Quantitative analysis of 11A50–B10^+^Aβ_40_ immunoreactive (IR) area normalized to retinal thickness in cross-sections from a cohort of AD (*n = *17), MCI (*n = *8), and CN controls (*n = *11). **c** Schematic diagram for the region of interest (ROI) analyzed with separate assessments for inner (from inner limiting membrane = ILM to inner nuclear layer = INL) and outer neural retina (from outer plexiform layer = OPL to outer limiting membrane = OLM). **d** Quantitative analysis of Aβ_40_ IR area in outer (O) vs. inner (I) retina of AD (*n = *17), MCI (*n = *8), and CN (*n = *11) human donors. **e** Quantitative analysis of Aβ_40_ IR area in central (C), mid-peripheral (M), and far-peripheral (F) retina from the same human cohort. **f** Mapping of Aβ_40_ in four quadrants, C/M/F, and inner vs. outer retina. Strength of magenta pseudo-color represents the density of retinal Aβ_40_ burden in each geographic region. **g** Analysis of retinal parameters when samples are stratified per two diagnostic groups, MCI/AD and CN for total retinal Aβ_40_ (*n = *22 MCI/AD and *n = *10 CN). Dotted lines display the suggested values to separate between control and disease groups. Males in filled circles and Females in clear circles. **h–j** Pearson’s coefficient (*r*) correlation between retinal Aβ_40_ IR area against **h** neuritic Aβ plaques in whole brain (gray dots) and entorhinal cortex (EC, red dots), **i** CAA scores, and **j** mini-mental state examination (MMSE) cognitive scores (gray dots—all retina, red dots—temporal retina = mean of ST and TI quadrants) in different subsets of AD, MCI, and CN human donors (*n = *20, *n = *17 or *n = *10, respectively). Data from individual human subjects as well as group mean ± SEM are shown. Fold and percent changes are shown in red. *p < 0.05, **p < 0.01, ***p < 0.001, ****p < 0.0001, by one-way or two-way ANOVA with Sidak’s post-hoc multiple comparison test (Red * in **e** indicates AD vs. CN group, blue * in **e** indicates AD vs. MCI group). Two group statistical analysis of ELISA was done by unpaired 2-tailed Student’s t test

**Fig. 6:**
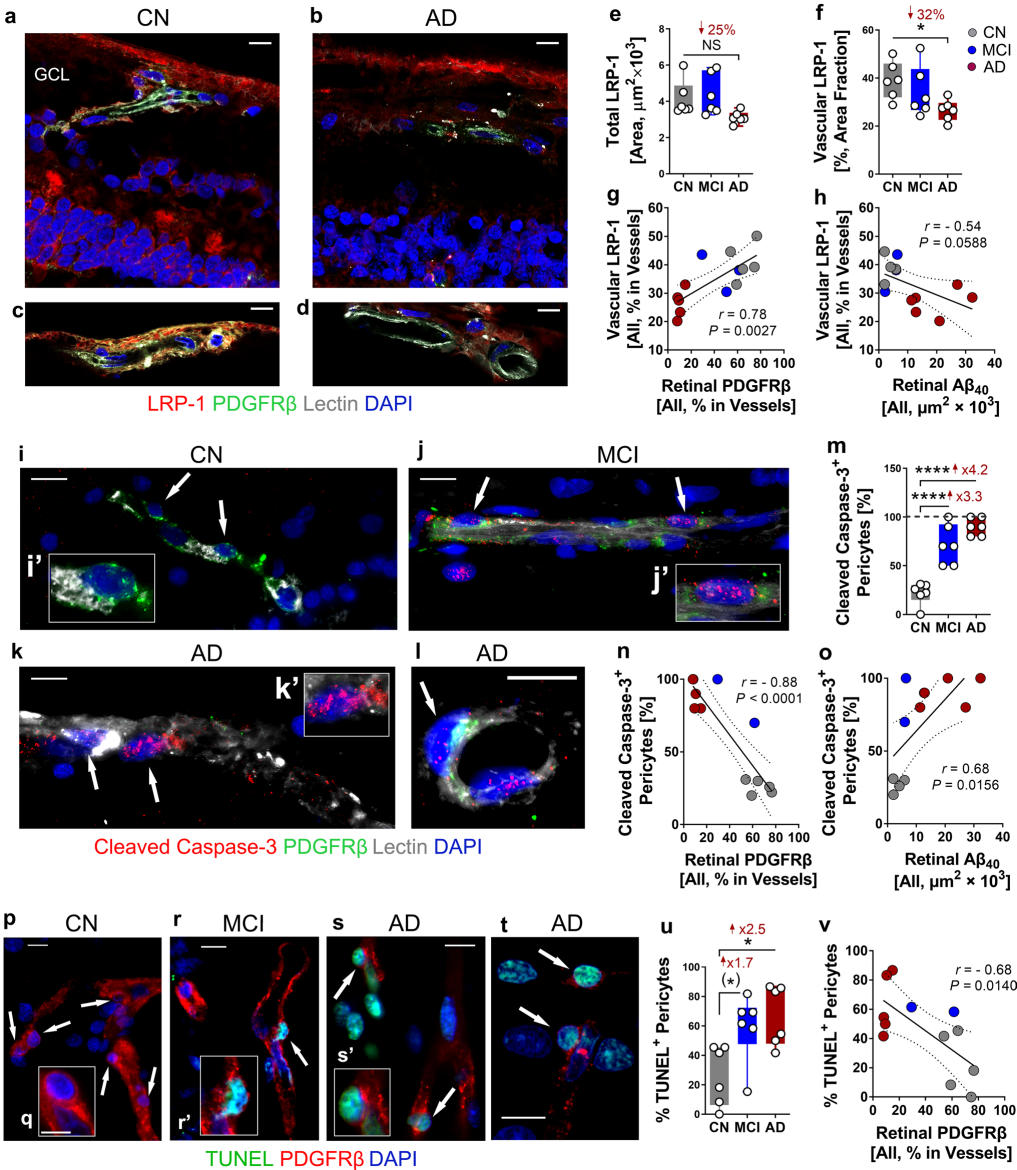
Decreased retinal LRP-1 in AD and increased apoptotic pericytes in MCI and AD retina. **a**, **b** Representative fluorescent images of paraffin-embedded retinal cross-sections isolated from **a** cognitively normal (CN) and **b** AD subjects, stained for LRP-1 (red), PDGFRβ (green), blood vessels (lectin, white), and nuclei (DAPI, blue). **c**, **d** Representative fluorescent images from AD and CN subjects focusing on retinal vascular LRP-1 region. **e** Quantitative analysis of total LRP-1 immunoreactive (IR) area in postmortem retinas from patients with AD (*n = *6), MCI (*n = *6), and from CN controls (*n = *6). **f** Quantitative analysis of percent LRP-1 IR area in retinal blood vessels from the same cohort. **g**, **h** Pearson’s coefficient (*r*) correlation between percent retinal LRP-1 IR area in the vasculature against **g** percent retinal vascular PDGFRβ IR area, and **h** total retinal 11A50–B10^+^Aβ_40_ area in a subset of human cohorts (*n = *13 and *n = *12, respectively). **i–l** Representative fluorescent images of paraffin-embedded retinal cross-sections isolated from **i** CN, **j** MCI, or **k**, **l** AD human eye donors, stained for cleaved caspase-3 (red), PDGFRβ (green), blood vessels (lectin, white), and nuclei (DAPI, blue). Arrows indicate positive signal of cleaved caspase-3 in pericytes. **i'**, **k’** show zoomed-in pericytes from the original image. **m** Quantitative analysis of percent cleaved caspase-3^+^ pericyte number out of 10–15 pericytes counted from each human donor: AD (*n = *6), MCI (*n = *6), and CN (*n = *6). Dashed line represents 100% reference point. **n**, **o** Pearson’s coefficient (*r*) correlation between percent cleaved caspase-3^+^ pericytes against **n** retinal vascular percent PDGFRβ IR area or **o** total retinal 11A50-B10^+^Aβ_40_ IR area in a subset of human donors (*n = *11). **p–t** Representative fluorescent images of paraffin-embedded retinal cross-sections isolated from human donors either **p**, **q** CN, **r** MCI, or **s**, **t** AD, stained for PDGFRβ (red), TUNEL (green) and nuclei (DAPI, blue). **r’**, **s’** show zoomed-in retinal TUNEL^+^ pericytes from the original images of MCI and AD donors. **u** Quantitative analysis of percent retinal TUNEL^+^ pericytes in 10–15 pericytes counted from each donor from the same human cohort. **v** Pearson’s coefficient (*r*) correlation between percent TUNEL^+^ pericytes and percent vascular PDGFRβ IR area in postmortem retinas from a subset of human donors (*n = *12). All scale bars = 10 µm. Data from individual human donors as well as group mean ± SEM are shown. Fold and percentage changes are shown in red. *p < 0.05, ****p < 0.0001, *NS* not significant, by one-way ANOVA with Sidak’s post-hoc multiple comparison test. *p < 0.05 in parenthesis = unpaired 2-tailed Student’s *t* test

For Figs. 2, 3, 4, 5, and 6 with analysis of retinal cross-sections and quantifications of PDGFRβ, vascular Aβ_42_, vascular Aβ_40_, Aβ_40_, LRP-1, cleaved caspase-3 and TUNEL, images were also acquired at the same setting and exposure time for each experiment, using the Axio Imager Z1 microscope, as described above. Images were captured at either 20 × or 40 × objectives, at respective resolutions of 0.5 and 0.25 µm. Three images were taken from central and far-peripheral retina and four images were taken from mid-peripheral retina (as shown in Fig. 2a, b). For each biomarker, the total area of immunoreactivity was determined using the same threshold percentage from the baseline in ImageJ (with same percentage threshold setting for all images), then subjected to particle analysis for each biomarker to determine their area or area percentage. For vascular PDGFRβ, vascular Aβ_42_ and Aβ_40_, and vascular LRP-1, area of blood vessels was chosen to acquire positive immunoreactive (IR) area percentage. For total retinal Aβ_40_ and total LRP-1 area, we chose the whole retina and documented total IR area of each biomarkers. Quantification of cleaved caspase-3^+^ and TUNEL^+^ pericytes was performed by randomly choosing 10–15 pericytes from each human donor, followed by manually counting using the grid in ImageJ. Then a percentage of cleaved caspase-3^+^ or TUNEL^+^ pericytes was calculated.

For vascular markers of Aβ_42_, Aβ_40_, and PDGFRβ, analysis was performed separately for longitudinal blood vessels and vertical blood vessels. Retinal cross-sections in this study were cut sagittally from flatmount strips, hence blood vessels were categorized by the shape of lectin stain: either as vertical blood vessels (≥ 10 µm in diameter) or longitudinal blood vessels (~ 10 µm in diameter). Note: for vertical blood vessels, the vascular wall area (determined by lectin) was selected for analysis, while excluding the blood vessel lumen. For longitudinal blood vessels, the total blood vessel including lumen and wall were selected for quantitative analysis. Dotted eclipse or rectangle frames were added to the representative images to highlight the area of quantification for both vertical blood vessels and longitudinal blood vessels.

### Statistical analysis

GraphPad Prism 8.1.2 (GraphPad Software) was used for analyses. A comparison of three or more groups was performed using one-way ANOVA followed by Sidak’s multiple comparison post-hoc test of paired groups. Groups with two independent variables/factors were analyzed by two-way ANOVA followed by Sidak’s multiple comparison test to further understand interaction between the two independent variables. Two-group comparisons were analyzed using a two-tailed unpaired Student *t* test. The statistical association between two or more variables was determined by Pearson’s correlation coefficient (*r*) test (Gaussian-distributed variables; GraphPad Prism). Pearson’s *r* indicates direction and strength of the linear relationship between two variables. Required sample sizes for two group (differential mean) comparisons were calculated using the nQUERY *t* test model, assuming a two-sided *α* level of 0.05, 80% power, and unequal variances, with the means and common standard deviations for the different parameters. Results are expressed as mean ± standard error of the mean (SEM). *P* value less than 0.05 is considered significant.

## Results

### Retinal pericyte loss along with vascular Aβ deposits including within pericytes in isolated microvasculature from postmortem retina of AD patients

To exclusively investigate the extent of retinal microvascular amyloidosis and possible pericyte degeneration in AD without interference from other retinal tissues, we enzymatically digested retinas, preserved solely the vascular network [51, 77], and subsequently conducted fluorescent immunostaining for blood vessels (lectin), PDGFRβ, and different types of Aβ (Fig. 1; extended data in Supplementary Fig. 2, online resource). Our modified method for human retinal vascular isolation and immunofluorescence is illustrated in Fig. 1a. This approach was performed on postmortem retinas isolated from a cohort of age- and sex-matched human subjects with AD diagnosis (avg. age 79.20 ± 10.9 years, 3 females/2 males, CAA score 1.7 ± 0.27) and CN controls (avg. age 75.60 ± 5.63 years, 2 females/3 males, no known CAA). Intense deposits of Aβ_42_ were visible in AD retinal microvasculature including colocalization with lectin, as compared to CN retina (Fig. 1b).

Vascular Aβ_42_ accumulation was also detected inside retinal pericytes in AD but not in CN (Fig. 1c). This is further supported by immunostaining with other antibodies against Aβ, including 11A50–B10 (Aβ_40_), 6E10, and 4G8 (Fig. 1d, e, Supplementary Fig. 2a–d, online resource). In addition, a substantial decrease in PDGFRβ expression was observed in retinal microvasculature from AD as compared to CN controls (Fig. 1f; see lectin/PDGFRβ co-labeling in yellow). A quantitative analysis of retinal microvascular pericyte count per microscopic visual field (1.8 × 10^4^ µm^2^) revealed a significant 37% pericyte loss in AD compared to controls (Fig. 1g). By quantification of Aβ-immunoreactive area normalized for the lectin-positive vascular area, a significant 5.6-fold increase of Aβ deposition in retinal microvasculature was measured in AD vs. CN (Fig. 1h). Moreover, a substantial 8.7-fold increase of Aβ-immunoreactive area within retinal pericytes was detected (Fig. 1i). Further, Aβ deposits were identified inside degenerated, acellular retinal capillaries that appear to lose lectin expression (Supplementary Fig. 2c, online resource).

Next, we validated the presence of Aβ deposition in isolated retinal blood vessel walls in double-transgenic murine models of AD (ADTg). Performing the blood perfusion procedure prior to retinal vascular extraction allowed us to exclude the contribution of circulating Aβ in the blood. A comparison between perfused and non-perfused ADTg mice and their non-transgenic littermates (WT) revealed that regardless of blood perfusion, there were substantial amounts of retinal vascular Aβ deposits in ADTg mice (Supplementary Fig. 3, online resource).

### Early and progressive loss of retinal vascular PDGFRβ is associated with CAA and brain amyloid plaque pathology

Retinal vascular pathology was further investigated in cross-sections prepared and analyzed from a larger cohort of 46 human eye donors with pre-mortem diagnosis of AD (*n* = 21), MCI (*n* = 11), or CN (*n* = 14). There were no significant differences in mean age, sex, or PMI between the three diagnostic groups (for more details see Supplementary Tables 1–2, online resource). Histological samples from this cohort were prepared through dissection of retinal strips (2 mm) from four quadrants (superior-temporal—ST, inferior-temporal—TI, inferior-nasal—IN, and superior-nasal—NS) spanning from the optic disc to the ora serrata, processed into paraffin-embedded cross-sections, and immunostained (Fig. 2a, b).

Initially, we assessed retinal vascular PDGFRβ expression by fluorescent immunostaining in lectin^+^ blood vessels. We classified and analyzed two types of blood vessels by shape and size: longitudinal (~ 10 µm in diameter) and vertical (≥ 10 µm in diameter). The examination of small-size longitudinal vessels allowed for analysis of PDGFRβ^+^ pericytes that exist in capillaries and pericytic venules, while excluding vSMCs in larger-size vessels. The separate analysis of vertical vessels covered both PDGFRβ-expressing pericytes and vSMCs. We observed a notable decrease of PDGFRβ signal in both retinal longitudinal and vertical blood vessels in MCI, which was further exacerbated in AD (Fig. 2c, d, Supplementary Fig. 4a, b, online resource). Stereological analysis of percent retinal PDGFRβ area in retinal cross-sections is shown in a subset of age- and sex-matched AD, MCI, and CN subjects (*n* = 38, avg. age ± SD: AD = 81.2 ± 15.3, MCI = 86.3 ± 6.2 and C*N = *78.1 ± 10.4). Data indicate a significant (38%) early loss of retinal PDGFRβ in vertical vessels of MCI as compared to CN controls, whereas a more profound reduction (72%) of vertical vascular PDGFRβ was detected in AD retina (Fig. 2e). To evaluate the relationship between retinal PDGFRβ and CAA scores, we applied Pearson’s correlation coefficient (*r*) analysis between the two parameters in a cohort of cognitively impaired individuals. We found a significant inverse relationship between retinal PDGFRβ levels and brain CAA score in MCI and AD (Fig. 2f), suggesting that retinal vascular changes in the form of PDGFRβ loss may predict amyloid angiopathy severity in the brains of these patients.

To measure changes in retinal PDGFRβ distribution across the four retinal quadrants, we analyzed PDGFRβ area coverage in human subjects that were stratified by their clinical diagnosis and for each quadrant separately (vertical vessels in Fig. 2g**–**j, longitudinal vessels in Supplementary Fig. 5a–e, online resource; for comparisons between the four quadrants see Supplementary Fig. 5g, online resource). Our analysis indicated that the temporal hemiretina (ST and TI) had early substantial decreases in vertical vascular PDGFRβ in MCI (Fig. 2i, j), whereas the percentage of PDGFRβ area loss was only significant at later disease stages in the nasal hemiretinal quadrants (NS and IN), as seen in AD (Fig. 2g, h). Consistent with the vascular impairment seen in vertical vessels, early and progressive loss of PDGFRβ^+^ pericytes residing along longitudinal capillaries and post-capillary venules was detected in MCI and AD (Supplementary Fig. 5a–e, online resource). A significant inverse association with neuropathological CAA scores was also identified among individuals with MCI and AD (Supplementary Fig. 5f, online resource).

Moreover, in subjects with neuropathological reports (*n* = 20), retinal PDGFRβ loss inversely correlated with brain Aβ plaques (NP, DP, IP) and NTs, as summarized in Fig. 2k, l heat-map (extended data on Pearson’s *r* correlations for pre-defined brain regions in Supplementary Table 3, online resource). In particular, significant correlations between retinal vascular PDGFRβ and brain neuritic plaques were detected for overall brain severity score, as well as separately for the hippocampus, entorhinal cortex, and visual association cortex (Fig. 2k, l). In a subset of subjects where MMSE scores were available, we identified a significant correlation between retinal PDGFRβ loss and cognitive impairment (Fig. 2m). While PDGFRβ in most of retinal quadrants significantly correlated with MMSE scores, the most significant correlation was found with the superior retina (Fig. 2m; extended data on Pearson’s *r* correlations between MMSE scores against retinal PDGFRβ, per each retinal subregion, are provided in Supplementary Table 5, online resource).

### Accumulation of retinal Aβ_42_ in blood vessels and pericytes in MCI and AD

Given that our group and others have demonstrated the existence of retinal Aβ deposits in AD patients [3, 25, 46, 47, 50], our next question was whether vascular PDGFRβ loss is associated with increased vascular Aβ deposition in postmortem retinas from MCI and AD patients. To this end, we studied retinal vascular Aβ_42_ pathology in a cohort of age- and sex-matched human eye donors (*n* = 31, avg. ± SD age: AD = 82.8 ± 18.4, MCI = 87.8 ± 5.5, CN = 78.8 ± 10.3; Fig. 3). We analyzed percent 12F4^+^Aβ_42_-IR area separately for vertical and longitudinal retinal vessels (Fig. 3a–d); the two types of blood vessels were classified as detailed above. The AD retina displays substantially more vascular Aβ_42_ as compared to both MCI and CN retinas (Fig. 3a–b; additional image panels in Supplementary Fig. 6a, b, online resource). In our quantitative IHC analyses, to avoid signal from circulating blood Aβ, in the vertical vessel analyses we quantify the immunoreactive area by selecting the vascular wall region and excluding the lumen area (see example of dotted eclipse frames in Fig. 3a). Analysis of vertical vascular Aβ_42_ confirmed a significant increase in the retina of MCI and AD compared to CN controls (Fig. 3c). Analysis of retinal longitudinal vessels also indicated a significant increase in Aβ_42_ burden in AD compared to MCI and CN controls (Fig. 3d), representing accumulation of both circulating and vascular Aβ_42_.

Next, investigation of the potential association between retinal vascular Aβ_42_ burden and respective CAA scores suggested a significant correlation, albeit in a limited cohort (Supplementary Fig. 6c, online resource). Additionally, retinal vascular Aβ_42_ load had a significant correlation with cerebral Aβ plaque burden (Fig. 3e), and moreover, a strong, inverse correlation with retinal PDGFRβ (Fig. 3f). Figure 3g demonstrates the gradual PDGFRβ loss concomitant with increased Aβ_42_ burden in retinas isolated from MCI and AD patients relative to CN controls (for extended representative images see Supplementary Fig. 7a–f, online resource**)**. Higher magnification fluorescent images show Aβ_42_ deposits inside residual retinal vascular PDGFRβ^+^ cells, with increased co-localization in MCI vs. AD (Fig. 3h; extended representative images in Supplementary Fig. 6d, e, online resource). TEM analysis in retinal vertical sections from AD patients reveals Aβ_42_ deposits in multiple locations near blood vessels and within pericytes (Fig. 3i, j). Retinal Aβ_42_ deposits were found perivascular in close proximity to pericytes (Fig. 3i, left), in the lumen adjacent to an endothelial cell (Fig. 3i, right), and inside pericytes (Fig. 3j).

### Substantial accumulation of vascular Aβ_40_ in AD retina

Since Aβ_40_ is the major alloform type deposited in cerebral blood vessels [34], we further studied its distribution in retinal blood vessels in a cohort of eye donors from age- and gender-matched individuals with diagnosis of AD, MCI, or CN (*n* = 36, avg. age ± SD: AD = 81.8 ± 14.8, MCI = 86.3 ± 6.2 and C*N = *78.1 ± 10.4; see Fig. 4 and extended data in Supplementary Figs. 8, 9, online resource). Initial analysis of vascular Aβ_40_ burden in paired retinas and brains utilizing peroxidase-based DAB staining with a specific antibody recognizing the C-terminal amino acid sequence of Aβ_40_ (JRF/cAβ40/28; courtesy of Janssen Pharmaceutica) revealed an increase in retinal vascular Aβ_40_ deposition in MCI and AD compared to CN controls (Fig. 4a–c). This was in agreement with our findings following application of a commercially available antibody (11A50–B10) recognizing the C-terminal sequence of Aβ_40_ (Supplementary Fig. 8a, online resource). Intriguingly, strong signal of Aβ_40_ was observed in the tunica media (Fig. 4c and Supplementary Fig. 8a, online resource), although deposits were also detected in tunica adventitia and intima (Fig. 4b, c and Supplementary Fig. 8a, online resource, see arrows).

Immunofluorescent staining using 11A50-B10 and JRF/cAβ40/28 antibodies demonstrated an extensive retinal vascular Aβ_40_ burden in both vertical and longitudinal vessels in AD (Fig. 4d–f; extended representative images in Supplementary Figs. 8b and 9a, b, online resource). Quantitative analysis of vascular 11A50–B10^+^Aβ_40_ immunoreactivity indicated substantial 7- to 12-fold increases in retinal vertical (vessel wall with lumen area excluded) and longitudinal vessels (representing both Aβ in circulating blood and vessel walls) in AD compared to CN (Fig. 4g, h). A non-significant trend was noted in MCI vs. CN controls and between MCI and AD groups. In an additional subset of human donors, analysis of retinal vascular Aβ_40_ using JRF/cAβ40/28 antibody verified significant increases in retinas from MCI and AD compared to CN controls (*n* = 14; Supplementary Fig. 6c, online resource).

Similar to retinal vascular Aβ_42_, retinal vascular Aβ_40_ burden significantly and inversely correlated with retinal PDGFRβ (Fig. 4i) and also tightly and directly correlated with retinal vascular Aβ_42_ burden (Fig. 4j). Moreover, retinal vascular Aβ_40_ was associated with entorhinal cortex parenchymal CAA in a subset of human donors where CAA scores are available (Supplementary Fig. 8d, online resource). Notably, increased retinal vascular Aβ_40_ burden positively correlated with elevated brain neuritic plaques (NPs), especially in the entorhinal cortex (Fig. 4k, l; for more details see Supplementary Table 4, online resource). The strongest correlation was observed with NTs in the visual association cortex (A-18). In addition, immature plaques in the primary visual cortex significantly associated with retinal vascular Aβ_40_ (Fig. 4k, l; Supplementary Table 4, online resource). Similar to vascular Aβ_42_, vascular Aβ_40_ was also detected in PDGFRβ^+^ cells (Supplementary Fig. 8e, online resource). Stratification of human subjects based on clinical AD/MCI vs. CN diagnosis revealed a significantly lower PDGFRβ and significantly higher Aβ_40_ and Aβ_42_ levels in AD/MCI retinal vessels (Fig. 4m**–**o). These findings highlight the potential to distinguish between the diagnostic groups using retinal vascular parameters, and especially vascular PDGFRβ and Aβ_40_ (Fig. 4n).

### Mapping retinal Aβ_40_ spatial and layer distribution in AD shows high burden in inner retinal layers from central regions

To evaluate the overall retinal Aβ_40_ burden, including abluminal deposits outside blood vessels, we quantified Aβ_40_ levels and mapped Aβ_40_-IR area in all four quadrants (ST, TI, IN, NS), central/mid/far (C/M/F) geometrical subregions, and inner vs. outer cellular layers of the neurosensory retina (Fig. 5). We initially measured retinal Aβ_1–40_ peptide levels in protein homogenates isolated from fresh-frozen donor eyes in an additional cohort of age- and gender-matched subjects (*n* = 11; 6 AD human eye donors: avg. ± SD age = 79.33 ± 17.6 years, 4 females and 2 males, and 5 CN controls: avg. age 75.4 ± 4.93 years, 3 females and 2 males). Quantitative ELISA revealed a significant increase in retinal Aβ_1–40_ concentrations in AD compared to CN (Fig. 5a). Immunofluorescence staining in a larger cohort (*n* = 36) using 11A50–B10 antibody confirmed a substantial elevation of retinal Aβ_40_ load in AD compared to MCI and CN (Fig. 5b, data were normalized per retinal thickness; for raw data see Supplementary Fig. 10f, online resource). Separate analyses of retinal Aβ_40_ burden per quadrant indicated consistently higher Aβ_40_ load in AD compared to MCI and CN, especially in TI quadrant (Supplementary Fig. 10a–d, online resource, for normalized data per retinal thickness; Supplementary Fig. 10 g**–**j, online resource, for raw data). As expected, a significant positive correlation was noted between total Aβ_40_ and vascular Aβ_40_ in the human retina (Supplementary Fig. 10e, online resource).

Our current observation of Aβ_40_ distribution predominantly in the inner retina and previous studies describing inner retinal pathology in AD, including thinning or RGC degeneration [6, 13, 18], prompt our analysis of Aβ_40_ burden in inner vs. outer retinal layers. To this end, we separated the inner retina (from inner limiting membrane to inner nuclear layer) and the outer retina (from outer plexiform layer to outer limiting membrane), as illustrated in Fig. 5c. This analysis revealed that the vast majority of retinal Aβ_40_ deposition (> 90%) is found in the inner as compared to the outer layers across all diagnostic groups (Fig. 5d), with evidence for propagation to the outer retina (~ 5%) in AD (for extended data on Aβ_40_ mapping in inner vs. outer retina with a separate analysis for each retinal quadrant see Supplementary Fig. 11a**–**j, online resource). Further evaluation of Aβ_40_ immunoreactivity in retinal C/M/F subregions indicated a significantly elevated burden in the central- vs. far-peripheral retina of AD, with similar but non-significant trends of increase in MCI (Fig. 5e; see comparative analysis of the four quadrants in Supplementary Fig. 11 k, online resource). A summary of retinal Aβ_40_ burden analyzed in four quadrants, three geometrical subregions, and inner vs. outer layers is illustrated by a color-coded pie graph (Fig. 5f).

The feasibility to separate between the MCI/AD and CN clinical groups by total retinal Aβ_40_ burden was further assessed. This analysis showed some overlap between the populations but indicated a significantly greater than fivefold increase in the retina of AD/MCI vs. CN controls (Fig. 5g). Finally, to examine possible associations between total retinal Aβ_40_ burden and other retinal and brain parameters, Pearson’s (*r*) correlations were calculated (Fig. 5h**–**j; Supplementary Fig. 11l, online resource). These analyses demonstrated a significant inverse correlation with retinal vascular PDGFRβ and non-significant correlations with brain NP (Fig. 5h), CAA scores (Fig. 5i), and cognitive status by MMSE (Fig. 5j; for correlations with different retinal quadrants see Supplementary Table 6, online resource). Nonetheless, a significant association was detected between retinal Aβ_40_ burden and NP in the entorhinal cortex (Fig. 5h).

### Vascular LRP-1 downregulation in AD retina and retinal pericyte apoptosis in MCI and AD

In murine models of AD, vascular LRP-1 was shown to be expressed by pericytes, mediate the clearance of brain-parenchymal Aβ via blood vessels, and affect cerebral amyloid deposition [69]. To evaluate LRP-1 and vascular LRP-1 expression in the human retina, we analyzed retinal cross-sections from a cohort of 18 subjects with AD, MCI, and CN (*n* = 6 subjects per each diagnostic group). Representative microscopic images demonstrated reduced vascular LRP-1 expression along with marked vascular PDGFRβ loss in postmortem retinas from AD as compared with CN control (Fig. 6a–d; extended images for separate channels in Supplementary Fig. 12a, b, online resource). A quantitative IHC analysis indicated a non-significant trend of decreased total retinal LRP-1 immunoreactivity in AD compared to CN controls (Fig. 6e), with no difference between levels of LRP-1 in MCI vs. CN controls. Evaluation of vascular LRP-1 expression revealed a significant 32% decrease in AD compared to CN (Fig. 6f). Retinal vascular LRP-1 significantly correlated with retinal vascular PDGFRβ in this cohort (Fig. 6g), yet showed a non-significant trend of association with retinal Aβ_40_ burden (Fig. 6h).

To investigate whether the findings of retinal PDGFRβ and pericyte loss in MCI and AD are due to apoptotic cell death, we evaluated two markers of apoptotic cells in this cohort. First, we immunolabelled cleaved caspase-3 and investigated apoptosis of pericytes in small blood vessels (Fig. 6i**–**o). Representative microscopic images show a frequent occurrence of cleaved caspase-3^+^ in pericyte nuclei in postmortem retinas from MCI and AD patients as compared to CN controls (Fig. 6j**–**l vs. i; extended images for separate channels in Supplementary Fig. 13a–d, online resource). Quantification of cleaved caspase-3^+^ pericyte number confirmed an early retinal pericyte apoptosis in MCI, which was on average higher in AD (Fig. 6m). Cleaved caspase-3 in retinal pericytes inversely and strongly correlated with retinal PDGFRβ and positively with retinal Aβ_40_ burden (Fig. 6n, o). To further validate apoptosis of retinal pericytes during AD progression, fluorescent TUNEL assay was utilized on the same cohort (representative microscopic images in Fig. 6p**–**t; extended images for separate channels in Supplementary Fig. 14a–d, online resource**)**. Analysis of TUNEL^+^ pericyte count indicated increased apoptosis of retinal pericytes in MCI and more significantly in AD (Fig. 6u), and a significant inverse correlation with retinal PDGFRβ (Fig. 6v).

## Discussion

In this study, we identified cellular and molecular changes involved in retinal vascular pathology in AD. Elastase-based enzymatic digestion, isolation, and clearance of retinal vascular network was applied to prevent possible interference of abluminal retinal tissue. This approach revealed the localization of retinal Aβ deposits within blood vessels, measured their accumulation including within pericytes, and established retinal pericyte loss in postmortem retinas of AD patients. Using murine models of AD and comparing between isolated retinal blood vessels from perfused and non-perfused animals, we demonstrated accumulation of Aβ in blood vessels, regardless of circulating Aβ in the blood. In a larger cohort of human eye donors, we mapped and quantitatively assessed various AD-related vascular parameters, such as PDGFRβ expression and Aβ burden, in anatomically pre-defined retinal subregions and layers. In the analysis of vertical blood vessels, by avoiding Aβ signal in the lumen, which may have originated from blood circulation, we were able to detect increased retinal vascular Aβ_40_ and Aβ_42_ burden in AD. We also demonstrated the existence of retinal Aβ accumulation in three layers of blood vessel walls.

In this study, we identified early and progressive loss of pericytes and vascular PDGFRβ expression in postmortem retinas from MCI and AD patients. Deficient PDGFRβ expression in the AD retina was tightly linked with increased retinal vascular Aβ_40_ and Aβ_42_ burden, and, importantly, was associated with CAA severity scores, brain Aβ plaques, and cognitive status. Along with elevated vascular amyloid deposits, retinal blood vessel cells had reduced LRP-1 expression and retinal pericytes showed elevated apoptotic biomarkers (cleaved caspase-3 and TUNEL), suggesting that vascular retinal pericytes undergo apoptosis and may have impaired LRP-1-mediated Aβ clearance in the AD retina. Our findings of early and extensive Aβ-associated retinal vascular PDGFRβ^+^ pericyte degeneration in MCI and AD mirror a prominent feature of brain AD pathology [11]. This feature was implicated in progressive BBB abnormalities, including insufficient Aβ clearance and neuronal damage [23, 28]. Together with previous identification of Aβ deposits and p-tau in the retina of AD patients [3, 25, 37, 46–48, 50, 63], these novel retinal vascular findings further establish the retina as a tissue affected by AD. Given that the neurosensory retina is an extension of the brain and far more accessible for visualization via noninvasive imaging at sub-cellular resolution [2, 52], the current study is expected to contribute to the understanding of retinal vascular pathophysiology of AD and guide developments of next-generation retinal biomarker imaging for AD.

In our cohort, an early increase of retinal Aβ_42_ deposits in vertical vessel walls (with lumen exclusion) was detected in MCI as compared to CN controls. This result, together with vascular Aβ_42_ levels already notable in CN individuals, suggests early retinal vascular Aβ_42_ deposits in the AD continuum and perhaps less efficient clearance compared to retinal Aβ_40_. Although both Aβ alloforms exhibit increased trends in retinal blood vessels of MCI when compared to CN, the fold changes in vascular Aβ_40_ between AD vs. CN controls were substantially higher than the respective increases for vascular Aβ_42_. These data suggest that during AD pathogenesis, Aβ_40_ is more prominently elevated in retinal blood vessels than Aβ_42_. Future studies should evaluate which alloform, Aβ_40_ or Aβ_42_ in blood vessels, accumulates earlier in the retina and may affect vascular abnormalities related to AD.

Importantly, the correlations between both vascular Aβ alloforms and PDGFRβ loss were significant, with a stronger correlation to Aβ_42_, possibly due to increased Aβ_42_ toxicity to pericytes. In murine models of AD, brain Aβ_42_ was detected within pericytes and was associated with pericyte loss [55, 66]. Further supporting this idea is our observation that retinal Aβ_42_ in MCI and AD is found inside residual punctate-stained PDGFRβ^+^ pericytes. A similar phenomenon was described in cerebral pericytes which were involved in Aβ_42_ clearance [55]. Other evidence for deposition of Aβ in retinal pericytes was provided in this study from quantification of Aβ in pericytes of isolated retinal blood vessels and by utilizing TEM analysis on retinal vertical sections. These findings suggest that similar to the brain, retinal pericytes may be susceptible to Aβ_42_ toxicity and play a role in its clearance in the retina. Nonetheless, these phenomena with possible implications to retinal Aβ_40_ and Aβ_42_ clearance mechanisms are poorly understood and warrant future investigations.

Our results have shown early and intense apoptosis of pericytes, as well as a decrease in PDGFRβ expression in pericytes and vSMCs. Brain pericytes and vSMCs are critical in regulating blood flow and BBB integrity [71]. Since PDGFRβ is expressed by both pericytes and vSMCs [38, 71, 79], and its signaling pathway is crucial for regulating pericyte recruitment [10, 17], our results of PDGFRβ loss in postmortem retina from MCI patients suggest an early compromised vascular integrity during the AD continuum, similar to that found in the brain [11, 24, 81]. Previously, brain pericyte loss and BBB breakdown were reported in AD patients [66, 83]. Additionally, in PDGFRβ^F7/F7^ mice, PDGFRβ deficiency led to brain pericyte reduction, resulting in both microvascular disruption and loss [80]. While vSMC actin was found to be reduced in AD brains [30], another report demonstrated disrupted PDGFRβ signaling and pericyte loss in PDGFRβ^F7/F7^ mice with no vSMC loss [61]. In the current study, we noted loss of retinal PDGFRβ staining in both vertical and longitudinal blood vessels, suggesting that both pericytes and vSMCs are affected in AD. Based on the separate analysis of small-size longitudinal capillaries and post-capillary venules [71], our data indicate substantial retinal PDGFRβ losses in pericytes from MCI and AD. The analysis of vertical vessels suggested significant retinal PDGFRβ losses in both pericytes and vSMCs. Future studies should identify which type of retinal vascular cells, pericytes or vSMCs, are more susceptible to injury due to AD, assess their connection with cerebral vascular abnormalities, and evaluate their impact on blood–retinal barrier integrity.

Previous studies identified a LRP-1-dependent mechanism of cerebral Aβ_42_ clearance in both brain vSMCs [30] and pericytes [55]. Cerebral LRP-1-mediated Aβ_40_ and Aβ_42_ clearance through apolipoprotein E isoforms-specific mechanism was further identified for PDGFRβ^+^ pericytes [55, 66]. In addition, a reduction in LRP-1 levels was reported in AD brains along with significant decreases in cortical neurons and vascular structures [41, 70]. In our study, a significant decrease (32%) of vascular LRP-1 expression was detected in postmortem retinas from AD patients. Together with this significant decrease, the trend of correlation between retinal vascular LRP-1 reduction and retinal Aβ_40_ accumulation may implicate a compromised retinal LRP-1-mediated Aβ_40_ clearance. These findings warrant future exploration of whether LRP-1 loss occurs later in disease progression, as a result of Aβ deposition, pericyte degeneration, or other earlier vascular abnormalities in the AD retina.

In this study, we found that retinal vascular amyloid burden consists of Aβ_42_ and Aβ_40_ alloforms, which is comparable to CAA composition in AD and MCI patients. Although both Aβ_42_ and Aβ_40_ are involved in CAA development [40, 56], Aβ_40_ has long been known to be the main alloform [57], and its accumulation associates with CAA progression [4]. Hence, due to its primary involvement in vascular amyloidosis and its distribution in various retinal layers, we quantified and mapped the spatial and layer distribution of total retinal Aβ_40_ burden. Importantly, the existence of retinal Aβ_1–40_ peptide was validated by a highly sensitive and specific sandwich ELISA and its significant accumulation in the temporal hemiretina of AD versus CN controls was demonstrated. Moreover, elevated Aβ_40_ burden in blood vessels from AD donors was further confirmed by commercial and proprietary (JRF/cAβ_40/28_) monoclonal antibodies specific to the C-terminal amino acid sequence of Aβ_40_ peptides, detected by both fluorescent and non-fluorescent labeling methods. Our results in postmortem retinas from MCI and AD patients show that Aβ_40_ deposition is detected in three layers of the vessel wall: tunica intima, media, and adventitia. Overall, the increased retinal Aβ_40_ burden may suggest Aβ-mediated toxicity to vascular cells that could lead to complications similar to CAA, including vessel wall fragmentation and blood leakage. Future studies should address this possibility.

Here, we observed a sevenfold increase in total Aβ_40_ burden in postmortem retinas of AD patients as compared to CN individuals, which was comparable with the increase in vascular Aβ_40_ burden. The significant correlation between the two parameters suggests that retinal vascular Aβ_40_ burden may be an outcome of total retinal Aβ_40_ accumulation. The abundance of apoptotic cell markers, TUNEL and cleaved caspase-3, in the nuclei of retinal pericytes of both MCI and AD, and the correlations with PDGFRβ loss and Aβ_40_ burden, may indicate that some aspects of retinal vascular abnormality are linked with increased total Aβ_40_ burden in the retina. All four retinal quadrants exhibited significantly higher total retinal Aβ_40_ burden in the AD group compared to both MCI and CN groups, with the highest 9.7-fold increase observed in the TI quadrant. Further, levels of Aβ_40_ in central retinal subregions were significantly higher compared to those measured in retinal far-periphery of AD. Importantly, over 90% of Aβ_40_ burden was concentrated in the inner retina compared to the outer retina, with signs of propagation from inner to outer retina during disease progression. These data corroborate previous observations of frequent Aβ deposits in inner retinal layers of AD and may explain excessive degeneration seen in RGCs and RNFL, as detected by histology and OCT [6, 26, 47, 50, 72, 82]. The buildup of Aβ_40_ in the central and inner retinal layers follows the pattern of highly dense retinal blood vessels in these regions and strengthens the possible link between Aβ accumulation, toxicity, and blood vessel disruptions [44, 45]. In addition, the substantial loss of PDGFRβ, especially in the ST and TI quadrants that colocalized with retinal vascular amyloidosis, and previous corroborating data indicating significant abnormalities in the ST and TI regions, imply that inner cellular layers in the central temporal hemiretina are more susceptible to AD pathological processes [6, 26, 47, 50, 82].

Retinal vascular Aβ_40_, vascular Aβ_42_, and total Aβ_40_ parameters appeared to correlate significantly with retinal PDGFRβ loss, suggesting their independent role in pericyte/vSMC toxicity and that the loss of these vascular cells may have direct effects on Aβ clearance and its vascular accumulation. Unexpectedly, retinal vascular Aβ_42_ correlated significantly with CAA scores, whereas retinal vascular and total Aβ_40_ only showed trends of significance with CAA severity. The limitation of these correlations is that the neuropathological reports with CAA scores were available for a smaller subset of human donors. Nevertheless, these findings possibly point to shared mechanisms of retinal and cerebral vascular Aβ_42_ accumulation, but independent mechanisms of vascular Aβ_40_ accumulation in the retina. It is intriguing that both vascular alloforms significantly correlated with Aβ plaque burden in the hippocampus, entorhinal cortex, and visual cortex—brain regions highly impacted by AD. Further, our data indicated retinal vascular PDGFRβ and Aβ_40_ burden as leading parameters to distinguish between MCI/AD and CN diagnostic groups, suggesting they may predict AD status. Given the morphological and physiological similarities between the BRB and BBB [64, 75], the loss of PDGFRβ^+^ pericytes along with Aβ deposits in retinal microvasculature and the associations with CAA and cognitive status point to the connection between retinal and brain pathology in AD.

To summarize, this study identifies early and progressive pericyte loss, compromised PDGFRβ expression, and vascular Aβ accumulation in postmortem retina of MCI and AD patients along with their significant correlation to cerebral pathology and cognitive decline. These results extensively impact our knowledge on early signs of retinal vascular AD pathology and the potential implications of disease progression. Damaged BRB-mediated ocular metabolism and subsequent vascular leakage are pivotal pathogenic activities implicated in multiple retinal microvascular diseases such as diabetic retinopathy and age-mediated macular degeneration [20, 53]. Our data suggest that traditional retinal vascular disease-related BRB pathologies may also be vastly involved in the AD retina. The discovery of pathogenic Aβ deposits and early pericyte loss in retinal blood vessels of MCI and AD could shed light onto the pathophysiological mechanisms of vascular disruption, increased BRB permeability, insufficient blood supply, disrupted immune responses, and neuronal degeneration. In light of the recent advances in live imaging of retinal blood microvessels (OCT angiography) [26, 42, 62, 82], pericyte imaging using adaptive optics [68], and retinal amyloid imaging [35, 47], these results should lead to future development of noninvasive retinal vascular amyloid and pericyte imaging technologies to facilitate early screening and monitoring of AD.

## Electronic supplementary material

Below is the link to the electronic supplementary material.

Supplementary file1 (PDF 71261 kb)

## Data Availability

The data that support the findings of this study are available from the corresponding author, upon reasonable request.
