# Supplementary material for: Identification of early pericyte loss and vascular amyloidosis in Alzheimer’s disease retina
Source: Acta Neuropathol. 2020 Feb 10;139(5):813–36. doi: 10.1007/s00401-020-02134-w (PMC7181564; doi:10.1007/s00401-020-02134-w)
Supplement: Supplementary file 1 — Supplementary file1 (PDF 71261 kb) [file 401_2020_2134_MOESM1_ESM.pdf]

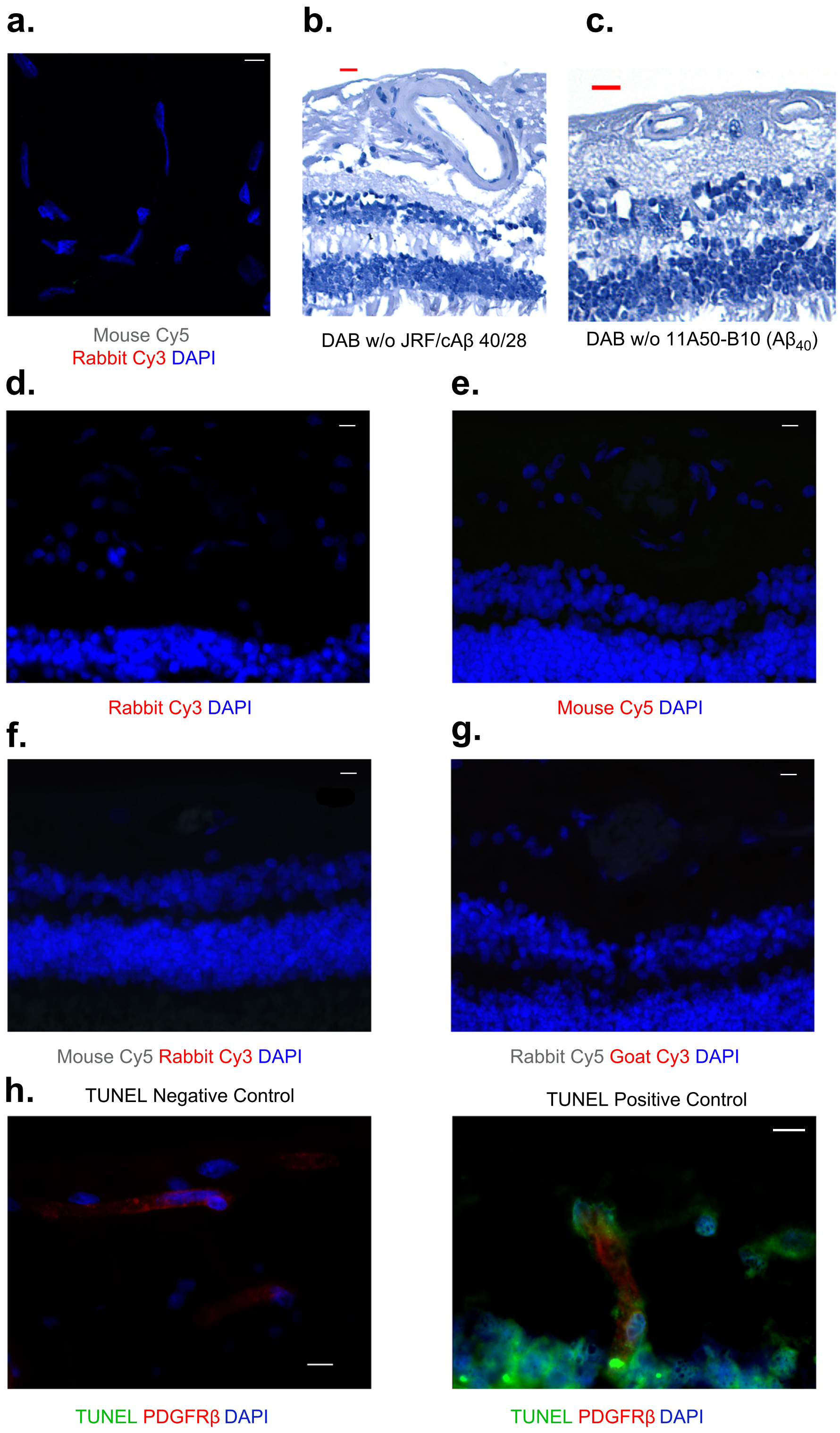

Supplementary Figure 1. Negative controls for all immunofluorescent staining. **a**. Representative images of negative controls (primary antibodies omitted) for isolated vascular staining in figure 1, with secondary antibodies (donkey anti-rabbit Cy3 and anti-mouse Cy5) and DAPI. Scale bar=10μm. **b-c**. Representative images of negative controls (primary antibodies omitted) for DAB staining without (w/o) **b**. JRF/cAβ 40/28 or **c**. 11A50-B10 ($A\beta_{40}$). Scale bars=20μm. **d**. Representative image of negative controls (primary antibodies omitted) for retinal cross-section staining in figure 2, with secondary antibody (donkey anti-rabbit Cy3) and DAPI. Scale bar=10μm. **e**. Representative image of negative controls (primary antibodies omitted) for retinal cross-section staining in figure 3 and 4, with secondary antibody (donkey anti-mouse Cy5) and DAPI. Scale bar=10μm. **f**. Representative image of negative controls (primary antibodies omitted) for retinal cross-section staining in figures 3 and 4, with secondary antibodies (donkey anti-rabbit Cy3 and anti-mouse Cy5) and DAPI. Scale bar=10μm. **g**. Representative image of negative controls (primary antibodies omitted) for retinal cross-section staining in figure 6, with second ary antibodies (donkey anti-goat Cy3 and anti-rabbit Cy5) and DAPI. Scale bar=10μm. **h**. Representative images of negative control (without termial transferase) and positive control (pretreatment with DNaseI) for TUNEL staining experiment in figure 6. Scale bars=10μm.

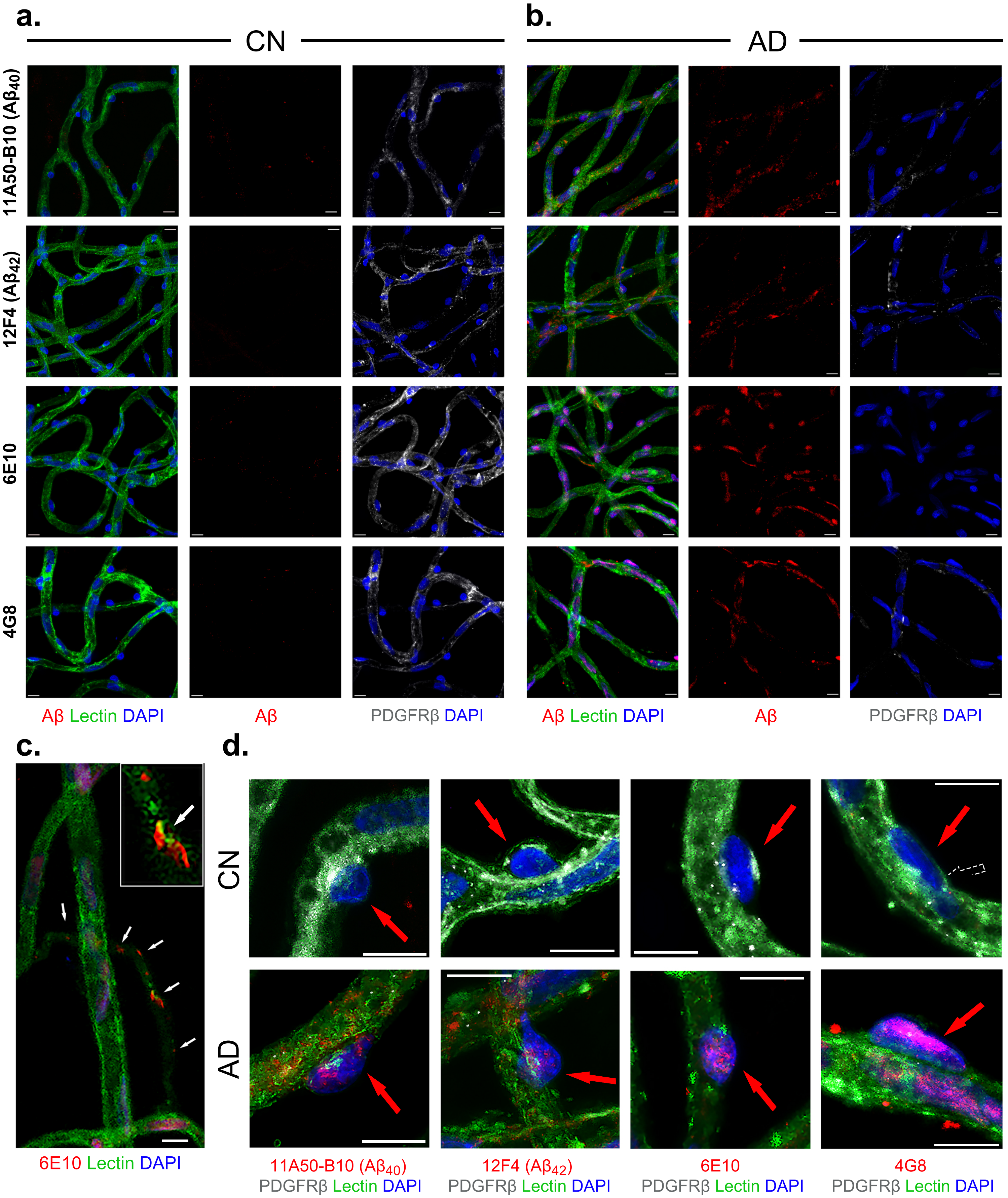

Supplementary Figure 2. Extended representative images for figure 1 on retinal microvascular Aβ and PDGFRβ[+] pericytes. **a-b**. Separate channels of representative fluorescent images for Aβ (11A50-B10, 12F4, 6E10 or 4G8 as indicated, red), PDGFRβ (pericytes, white), lectin (glycoprotein in blood vessel, green) and DAPI (nuclei, blue) on isolated retinal microvasculature from age and sex-matched human donors with AD (n=5) or cognitively normal (CN, n=5). **c**. Aβ (6E10, red), lectin (green) and DAPI (blue) staining showing Aβ deposits in a degenerated, acellular capillary (indicated by arrows). Upper right image shows zoomed-in image from the original. **d**. Enlarged confocal images of retinal microvascular pericytes from AD and CN donors (Aβ-red, PDGFRβ-white, lectin-green, DAPI-blue). Scale bars=10μm.

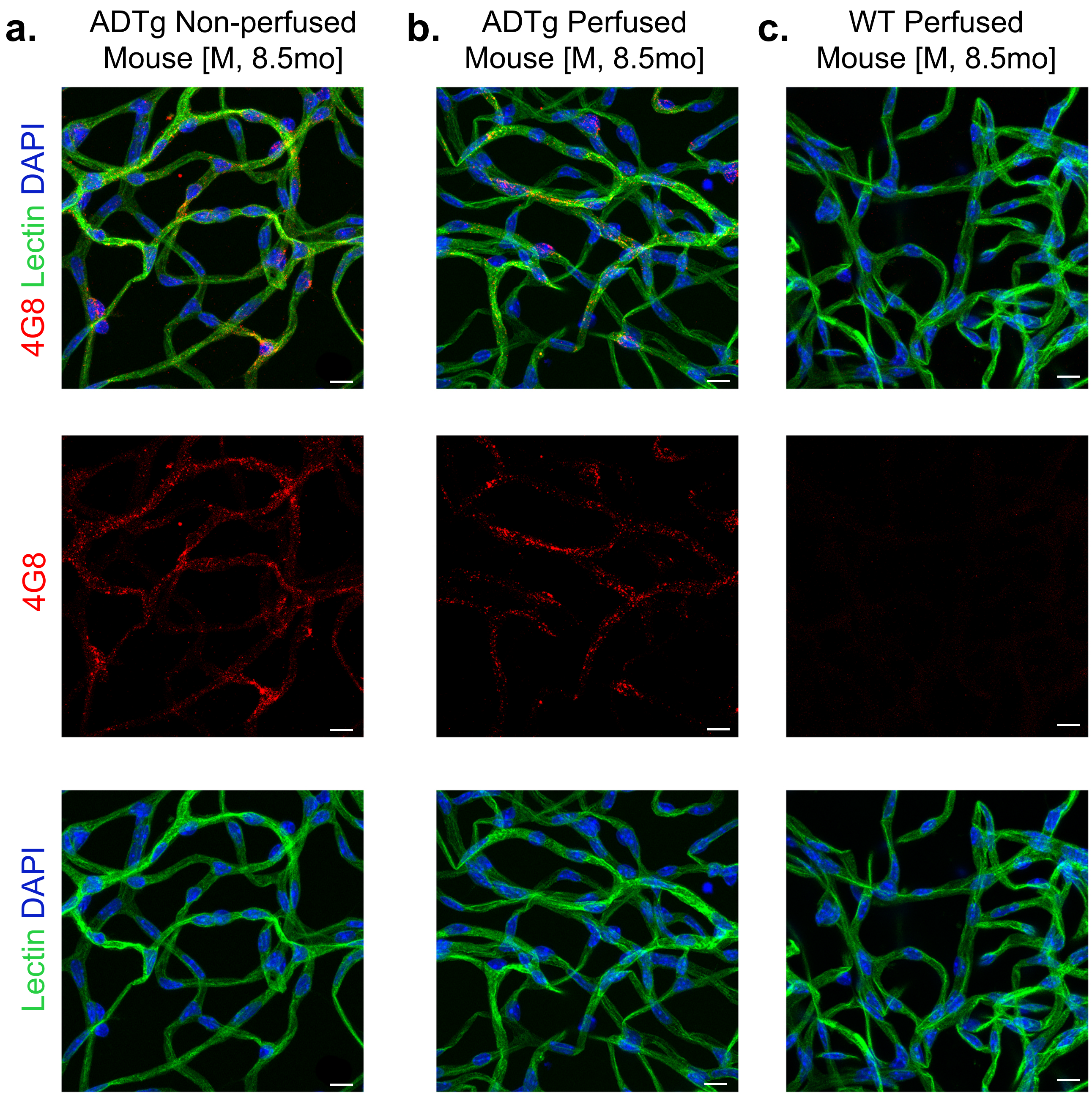

Supplementary Figure 3. Significant retinal vascular Aβ deposition in perfused transgenic ADTg mice. **a-c**. Representative fluorescent images for Aβ (4G8, red), lectin (glycoprotein in blood vessel, green) and DAPI (nuclei, blue) on isolated retinal microvasculature from **a**. non-perfused 8.5 month old male ADTg mice, or **b**. perfused 8.5 month old male ADTg mice, and **c**. perfused 8.5 month old male wild type mice. Scale bars=10μm.

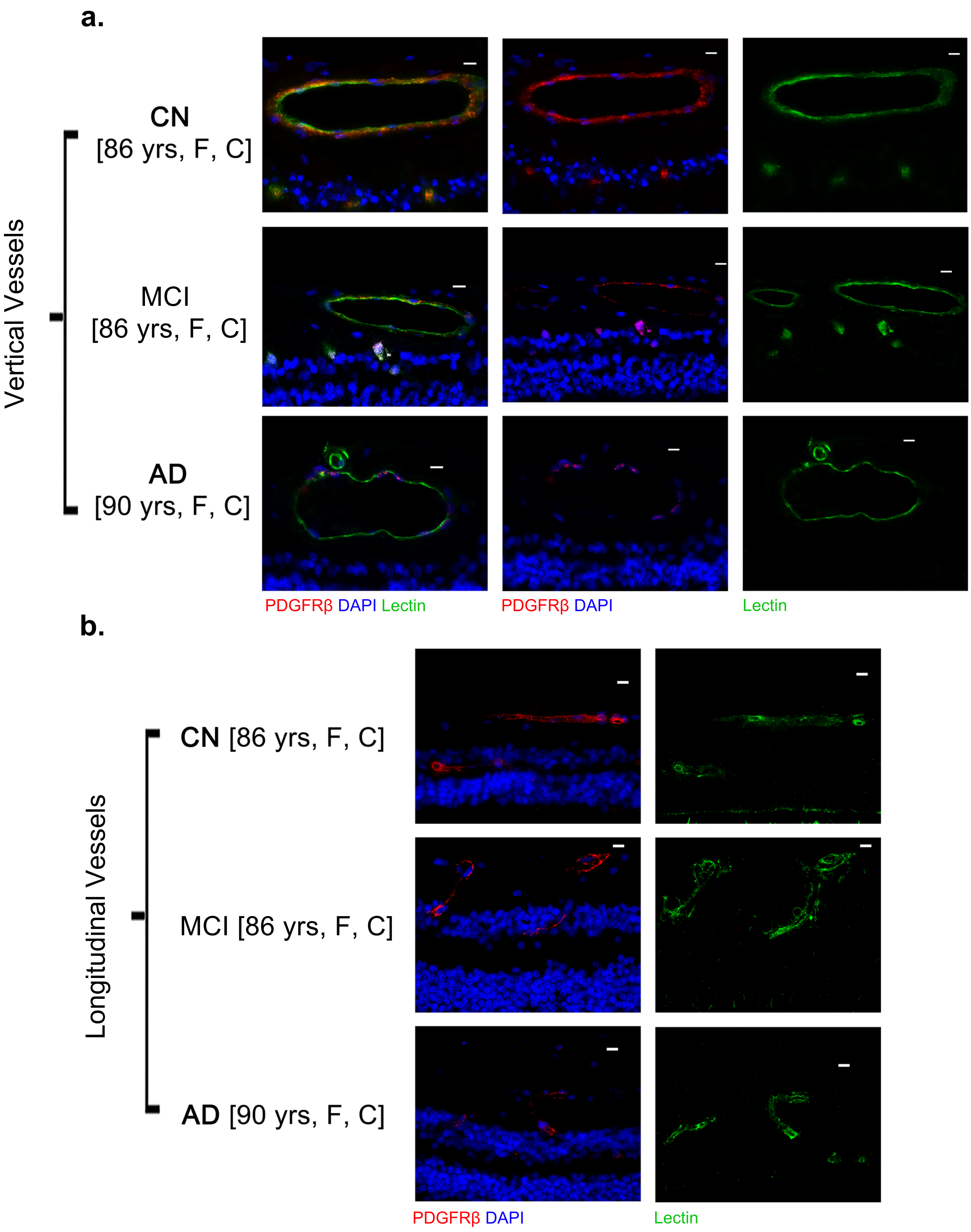

Supplementary Figure 4. Extended representative images for figure 2 on retinal vascular PDGFRβ. **a-b**. Merged and separate channels of representative fluorescent images for PDGFRβ (red), lectin (glycoprotein in blood vessel, green) and DAPI (nuclei, blue) in paraffin-embedded retinal cross-sections isolated from age and sex-matched human donors with AD, MCI or cognitively normal (CN). **a**. Vertical (V) and **b**. Longitudinal (L) blood vessels are shown. Scale bars=10μm.

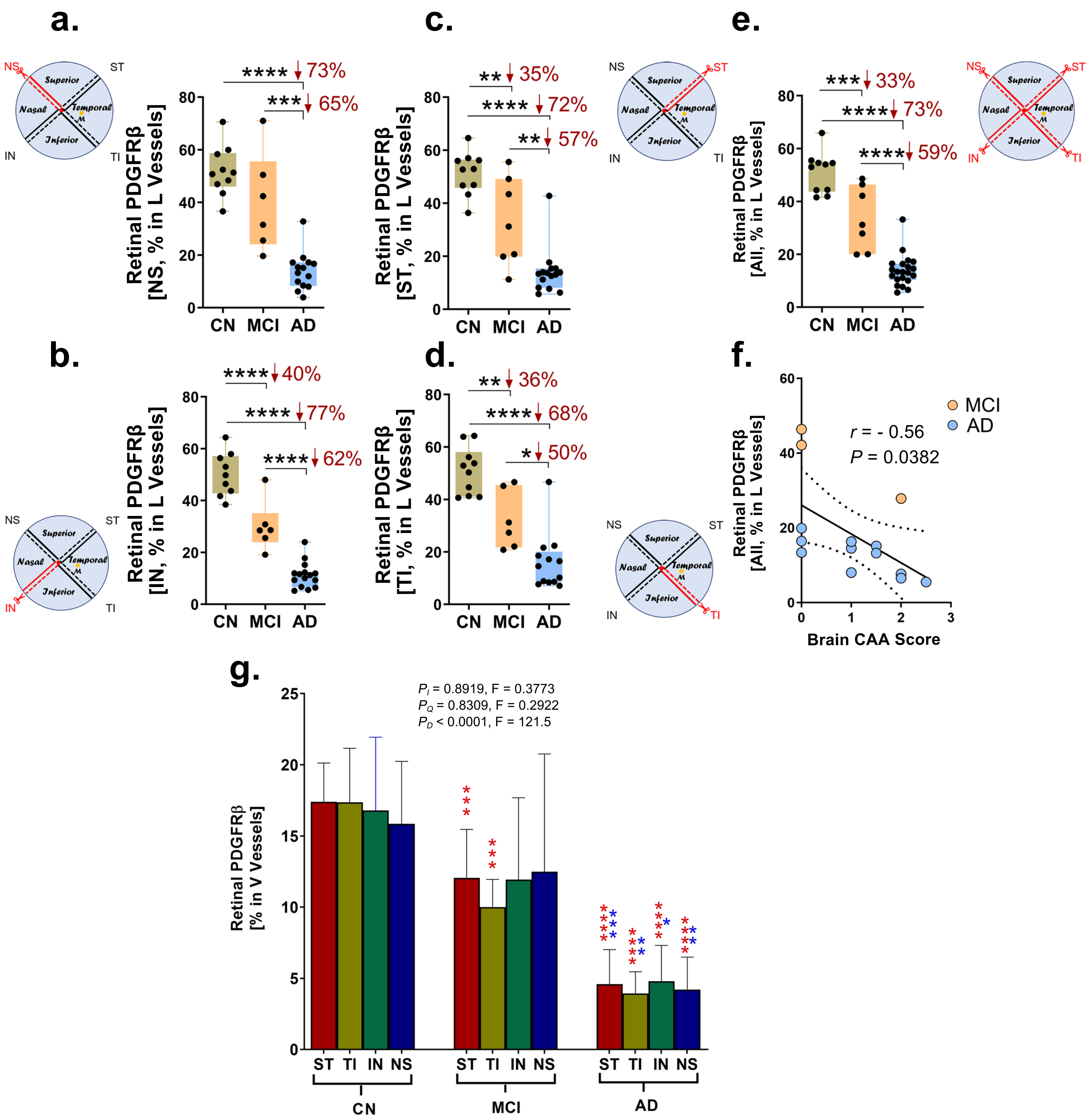

Supplementary Figure 5. Extended data on retinal PDGFRβ in longitudinal vasculature from all retinal quadrant regions in AD, MCI and CN human donors and mapping of PDGFRβ. **a-d**. Quantitative analysis of % PDGFRβ-immunoreactive area in longitudinal (L) vessels from each retinal quadrant separately: **a**. NS, **b**. IN, **c**. ST, **d**. TI in total cohort of AD (n=21), MCI (n=7) and cognitively normal (CN) (n=10) human donors. **e-f**. Quantitative analysis of retinal % PDGFRβ immunoreactivity in L vasculature (average of all four quadrants): **e**. subjects stratified by clinical diagnosis (n=38) and **f**. Pearson's correlations against brain CAA scores in a subset of this cohort (n=14). **g**. Mapping of vertical (V) vascular PDGFRβ in four retinal quadrants. (* indicates AD vs. CN, * indicates AD vs. MCI) Data from individuals as well as group means and SEMs are shown. *$p < 0.05$, **$p < 0.01$, ***$p < 0.001$, ****$p < 0.0001$, by one-way ANOVA test with Sidak's post-hoc multiple comparison test. Percent change are shown in red.

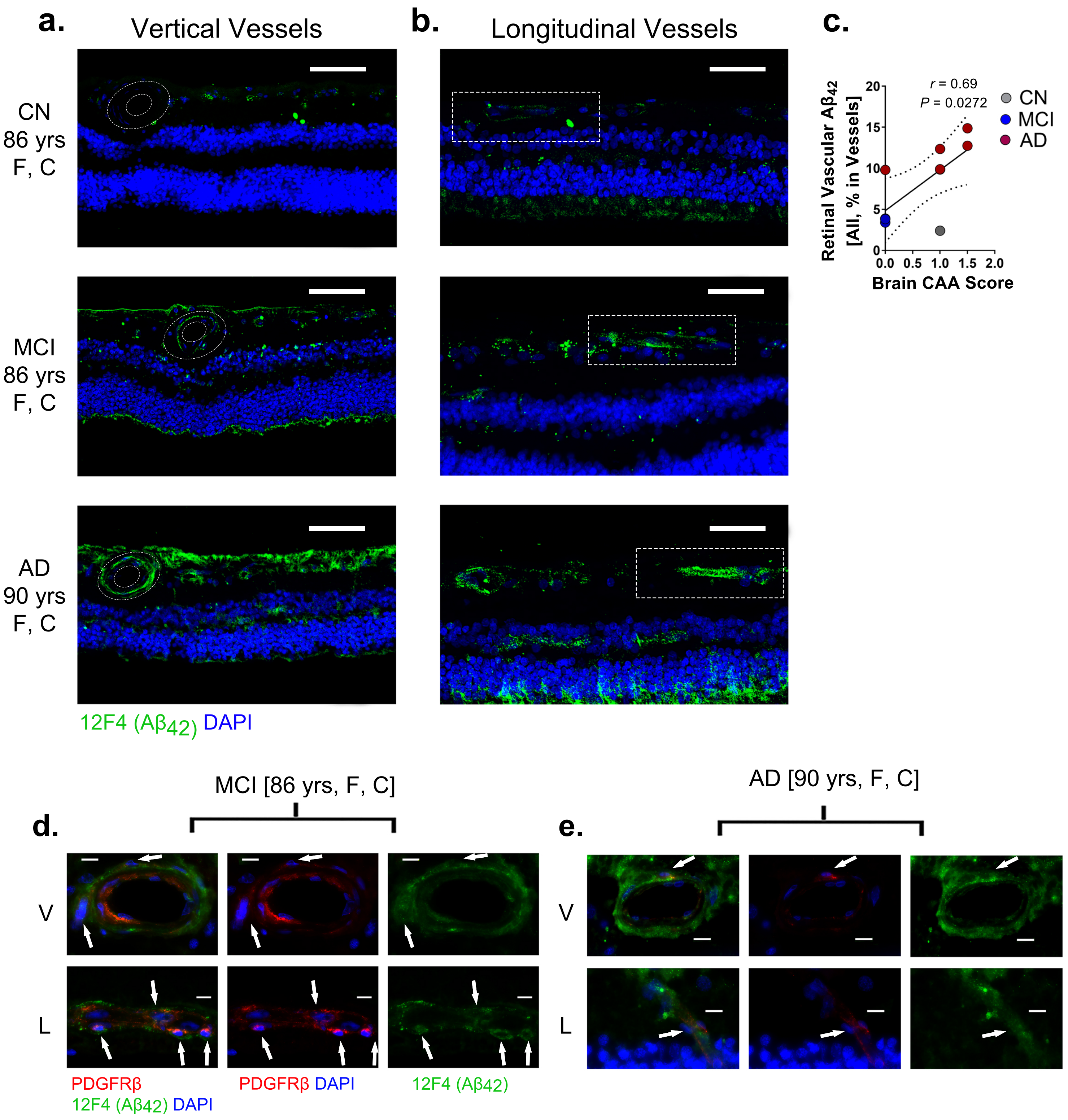

Supplementary Figure 6. Extended representative images for figure 3. **a-b**. Representative fluorescent images of paraffin-embedded retinal cross-sections isolated from age and sex-matched human donors with AD, MCI, or cognitively normal (CN) stained for $A\beta_{42}$ (12F4, green) and DAPI (nuclei, blue). **a**. Vertical (V) and **b**. longitudinal (L) blood vessels are shown. Dashed geometric shapes (white) indicate pre-defined areas of analysis. Scale bars=50 µm. **c**. Pearson's coefficient (r) correlation between retinal 12F4^{+} $A\beta_{42}$ burden in both V and L (All) blood vessels against brain CAA score. **d-e**. Merged and separate channels of representative fluorescent images for 12F4 ($A\beta_{42}$, green), PDGFRβ (red) and DAPI (nuclei, blue) in paraffin embedded retinal cross-sections isolated from age and sex-matched human subjects with AD and MCI. Arrows point at $A\beta_{42}$ in PDGFRβ^{+} cells. Both retinal V and L blood vessels in **d**. MCI and **e**. AD patients are shown (yrs=years old; F=female; C=Caucasian). Scale bars=10µm.

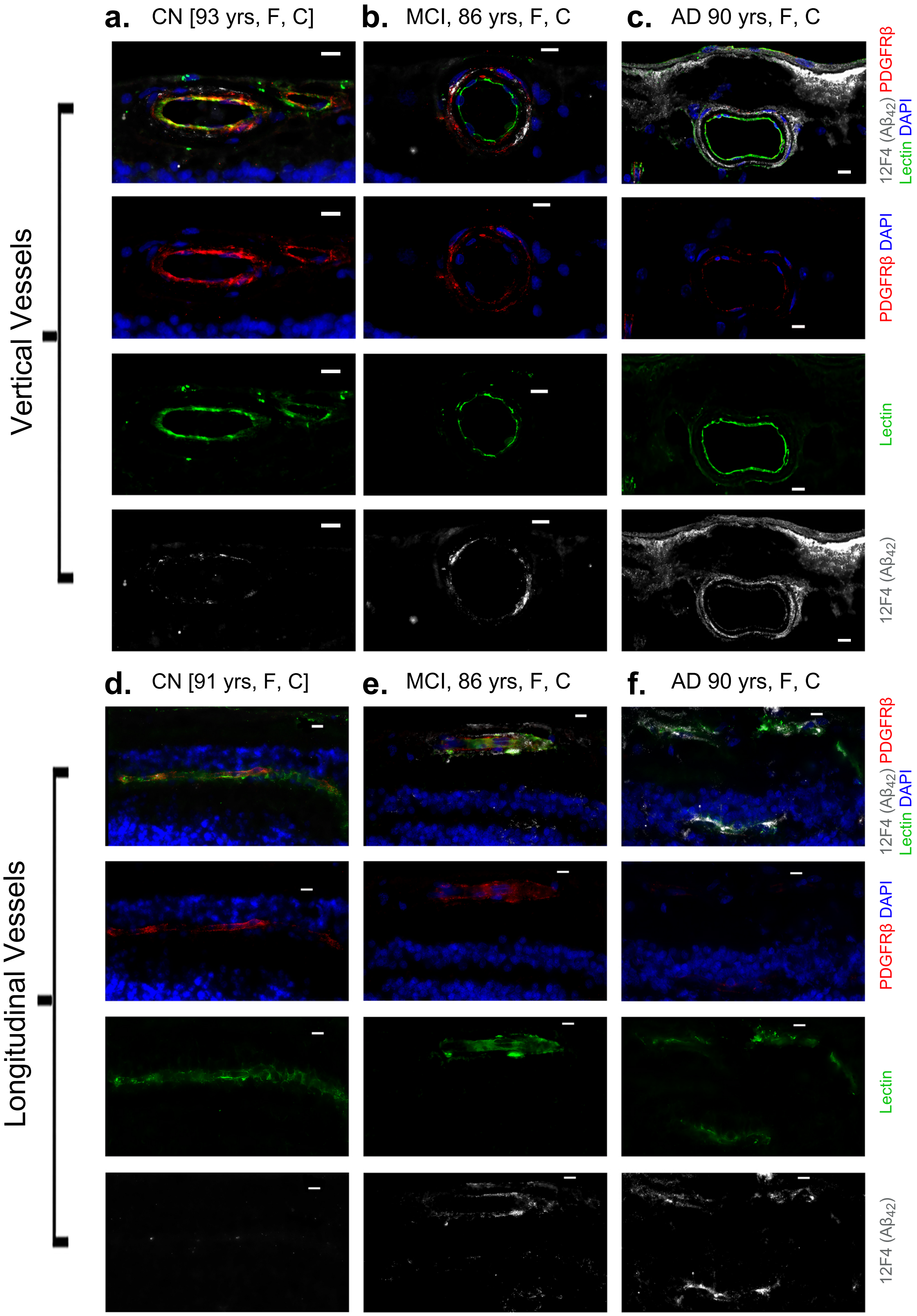

Supplementary Figure 7. Expanded representative images for Aβ_{42}, PDGFRβ, lectin and DAPI. **a-f**. Merged and separate channels of representative fluorescent images for 12F4 (Aβ_{42}, white), PDGFRβ (red), lectin (glycoprotein in blood vessel, green) and DAPI (nuclei, blue) in paraffin-embedded retinal cross-sections isolated from age and sex-matched human donors with AD, MCI or cognitively normal (CN). **a-c**. Vertical and **d-f**. Longitudinal vessels are shown (yrs=years old; F=female; C=Caucasian). Scale bars=10μm.

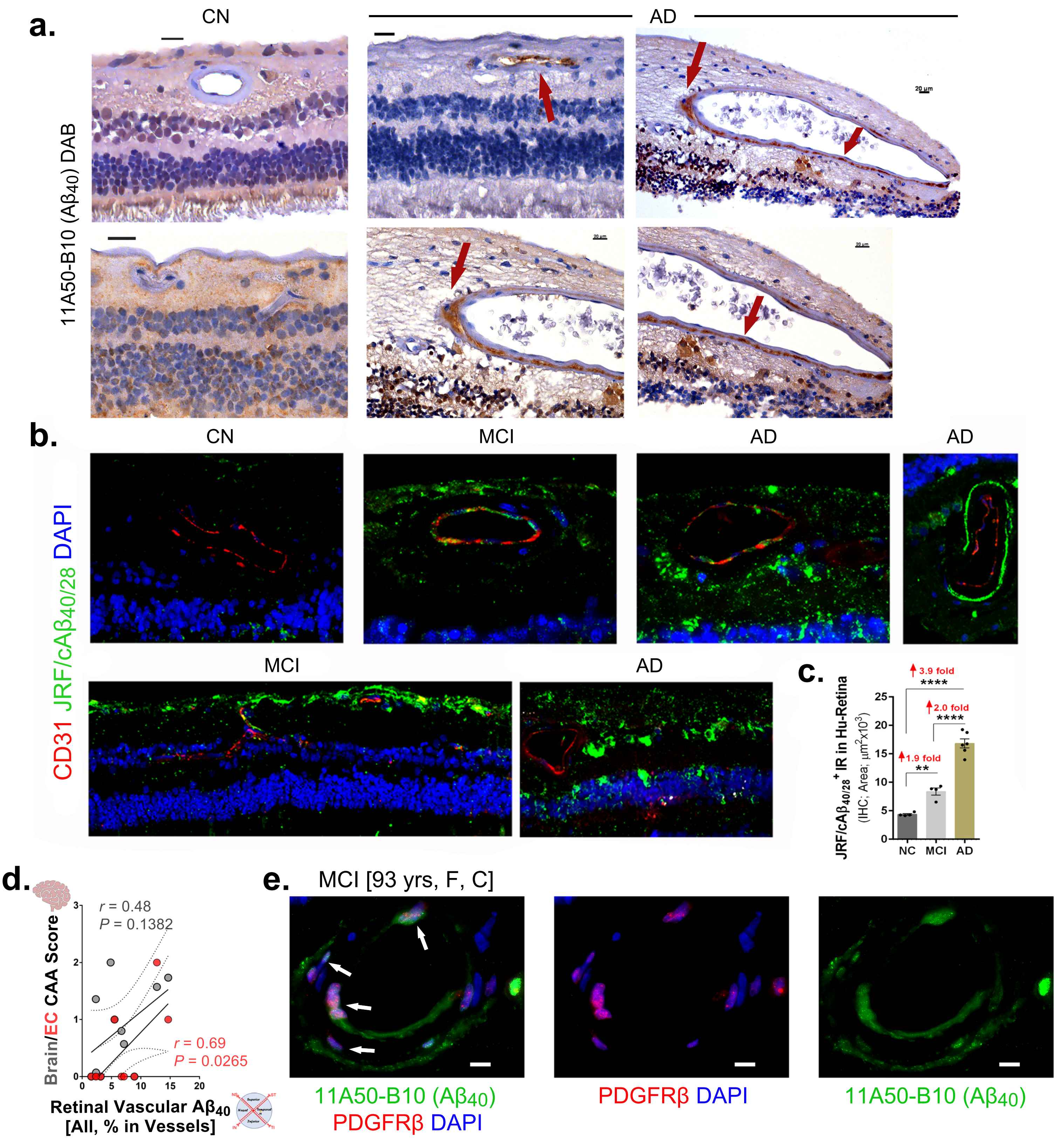

Supplementary Figure 8. Extended data on retinal vascular $A\beta_{40}$ deposits from MCI and AD human donors compared to cognitively normal controls. **a**. Representative horseradish peroxidase and 3,3'-Diaminobenzidine (DAB) staining of images for 11A50-B10-$A\beta_{40}$ in retinas from AD and cognitively normal (CN) subjects. Arrows indicate vascular $A\beta_{40}$ staining in tunica intima and media. Scale bars = 20 µm. **b**. Representative fluorescent microscope images of paraffin-embadded retinal cross-sections from AD, MCI and CN stained against $A\beta_{40}$ (JRF/cAβ 40/28 antibody; green), endothelial cells (CD31; red) and nuclei (DAPI, blue). **c**. Quantitative analysis of retinal vascular $A\beta_{40}$-IR area in a subset of MCI (n=4), AD (n=6) and CN (n=4) human donors. Data from individual human donors as well as group means and SEMs are shown. $^{**}p < 0.01$, $^{****}p < 0.0001$, by one-way ANOVA test with Sidak's post-hoc multiple comparison test. Fold change are shown in red. **d**. Pearson's coefficient (r) correlation between retinal $A\beta_{40}$ burden (11A50-B10-IR area) in both vertical and longitudinal vasculature (average) against CAA score in parenchymal brain average (grey) and entorhinal cortex (EC, red), within a subset of subjects with AD, MCI and CN (n=10). **e**. High-magnification images showing co-localization of vascular $A\beta_{40}$ (green) and PDGFRβ (red; co-localization indiacted by arrows) in a MCI subject (yrs=years old; F=female; C=Caucasian). Scale bars = 10µm.

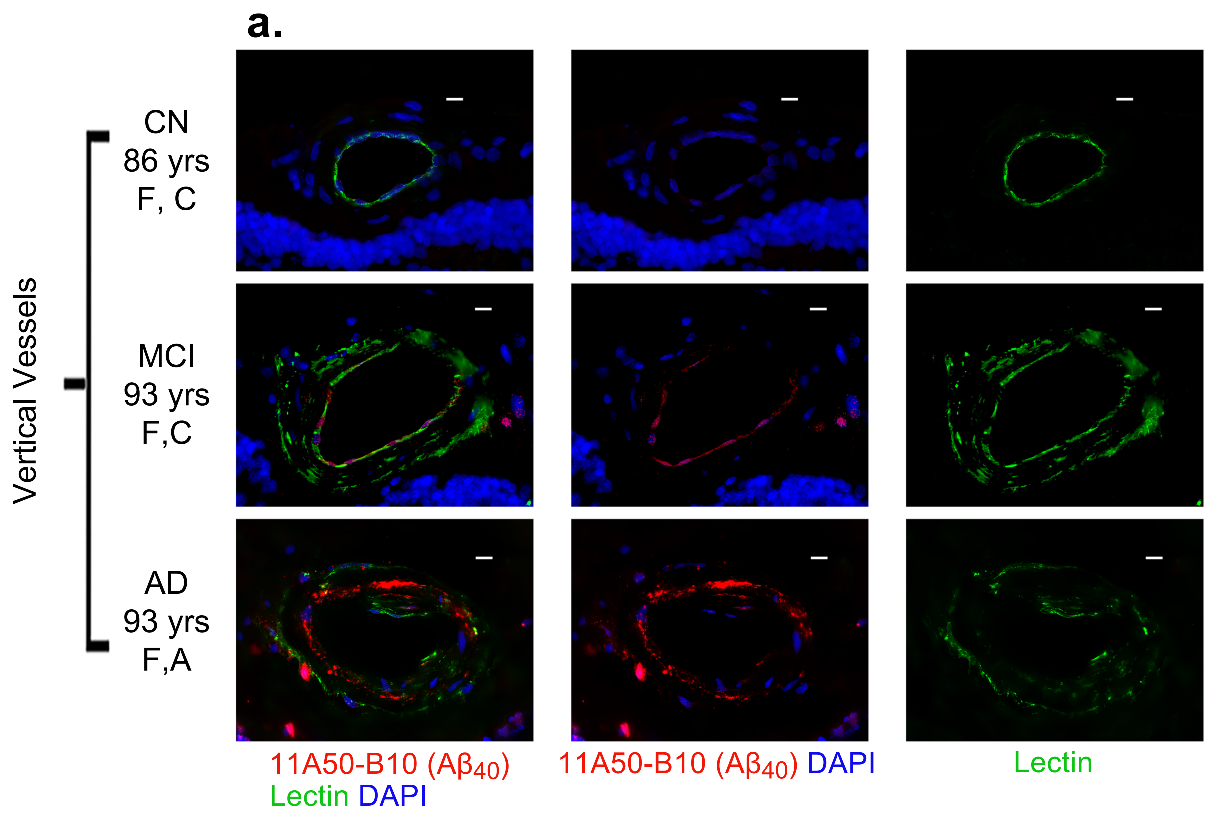

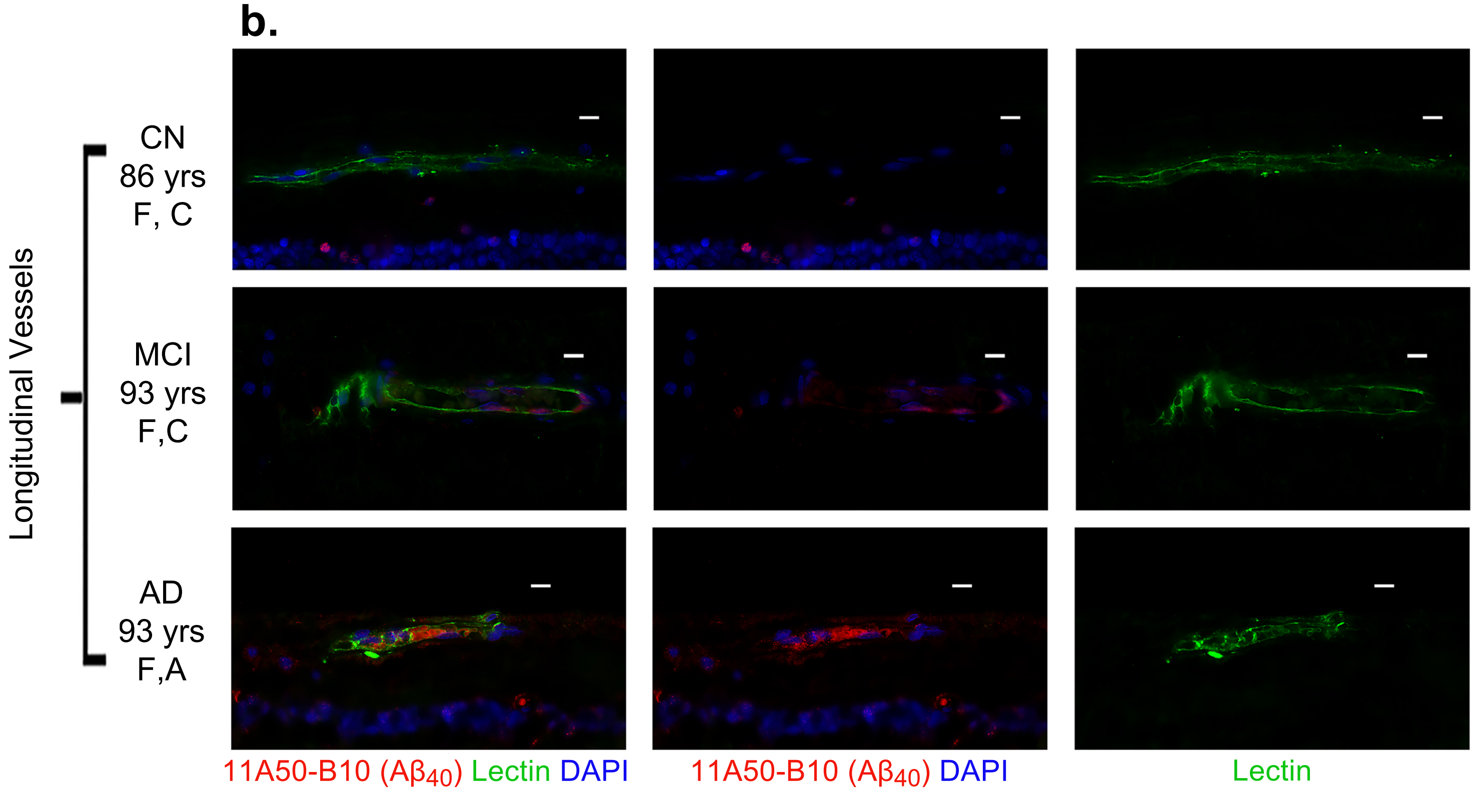

Supplementary Figure 9. Extended representative images for retinal Aβ_{40}. **a-b**. Merged and separate channels of representative fluorescent images for 11A50-B10 (Aβ_{40}, red), lectin (glycoprotein in blood vessel, green) and DAPI (nuclei, blue) in paraffin-embadded retinal cross-sections isolated from age and sex-matched human donors with AD, MCI or cognitively normal (CN). **a**. Vertical and **b**. Longitudinal vessels are shown(yrs=years old; F=female; C=Caucasian; A=Asian). Scale bars=10μm.

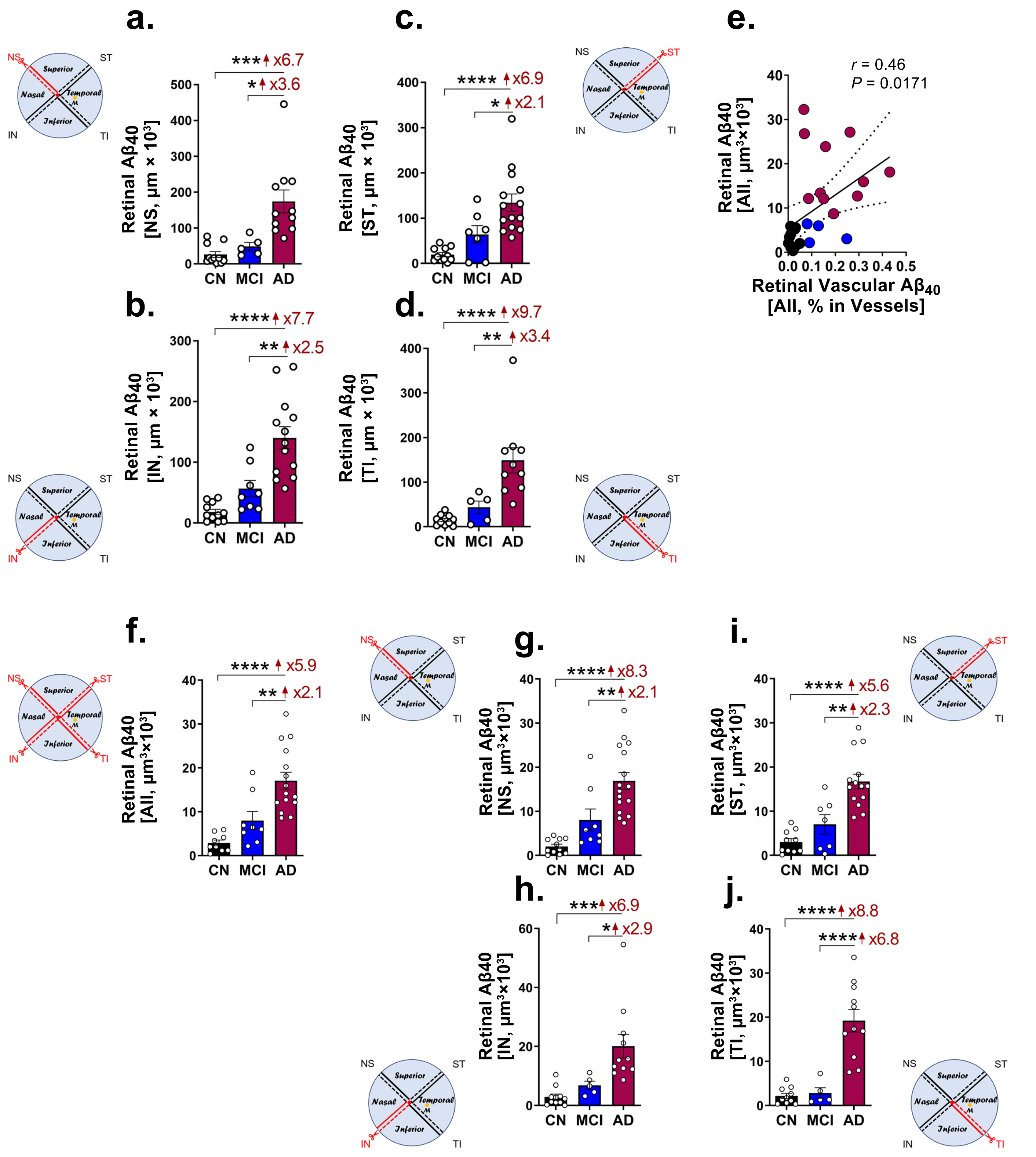

Supplementary Figure 10. Supplementary information for quantification of Aβ_{40} in human retina. **a-d**. Quantitative analysis of 11A50-B10 (Aβ_{40}) immunoreactive (IR) area (normalized by retinal thickness) in retinal layers (from inner to outer limiting membrane) from each retinal quadrant separately: **a**. NS, **b**. IN, **c**. ST, **d**. TI in AD (n=17), MCI (n=8), and CN (n=11) human donors. **e**. Pearson's coefficient (r) correlation between Aβ_{40} IR area and retinal Aβ_{40} burden in blood vessels (n=26). **f-j**. Quantitative analysis of raw data of 11A50-B10 (Aβ_{40})-IR area in retinal layers from each retinal quadrant separately and all quadrants together: **f**. all retinal quadrants, **g**. NS, **h**. IN, **i**. ST and **j**. TI in AD (n=17), MCI (n=8) and CN (n=11) human donors. *$p < 0.05$, **$p < 0.01$, ***$p < 0.001$, ****$p < 0.0001$, by one-way ANOVA test with Sidak's post-hoc multiple comparison test. Fold changes are shown in red.

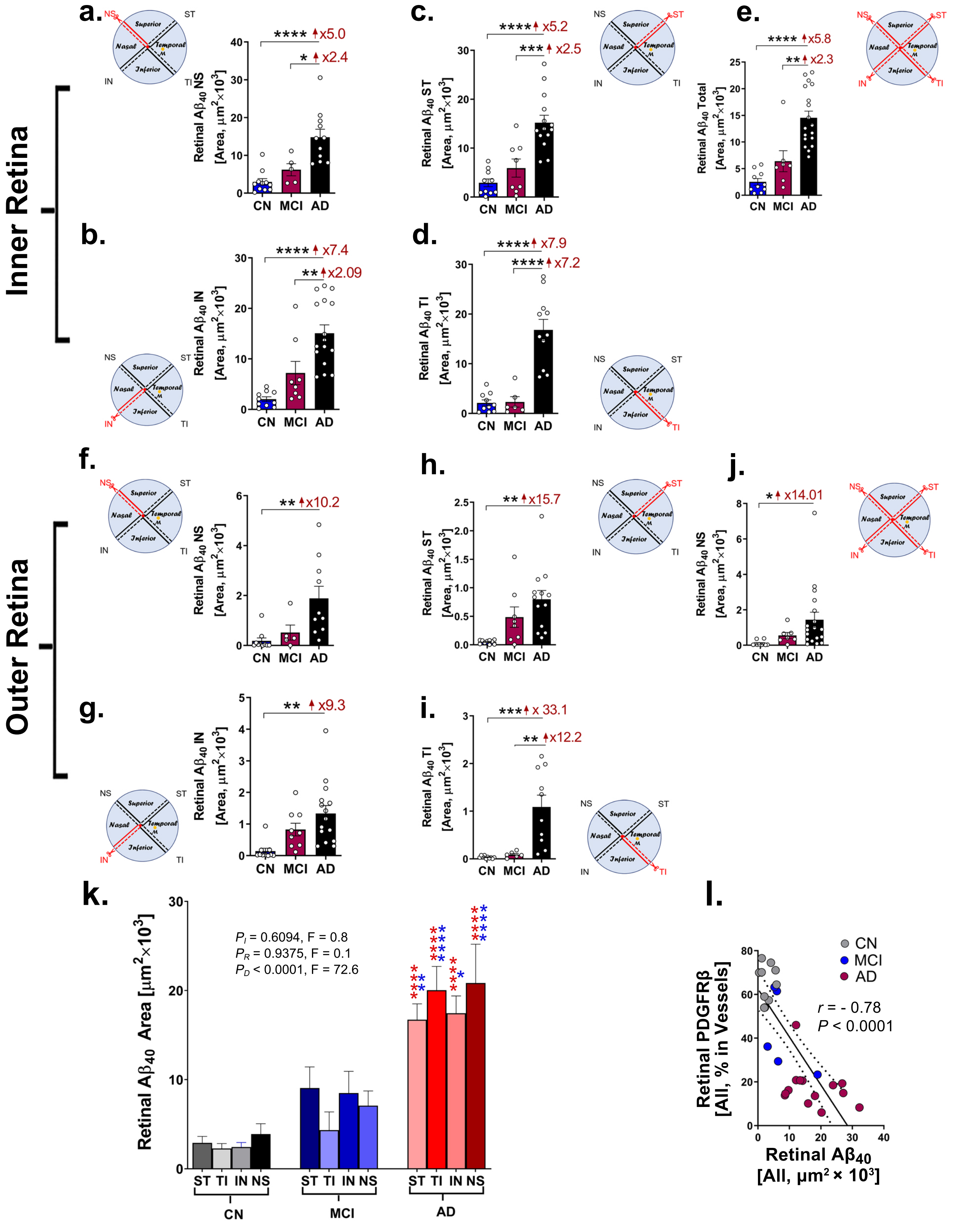

Supplementary Figure 11. Extended data of inner vs. outer retinal Aβ_{40} and mapping for all four retinal quadrants. **a-e**. Quantitative analysis of raw data of Aβ_{40}-immunoreactive (IR) area in inner retinal layers from each retinal quadrant separately and all quadrants together: **a**. NS, **b**. IN, **c**. ST, **d**. TI, **e**. all, in AD (n=17), MCI (n=8) and CN (n=11) human donors. **f-j**. Quantitative analysis of raw data of Aβ_{40}-IR area in outer retinal layers from each retinal quadrant separately and all quadrants together: **f**. NS, **g**. IN, **h**. ST, **i**. TI, **j**. all, in AD (n=17), MCI (n=8) and CN (n=11) human donors. **k**. Mapping of Aβ_{40} in four retinal quadrants (* indicates AD vs. CN, * indicates AD vs. MCI). **l**. Pearson's coefficient (r) correlation between Aβ_{40} IR area against % PDGFRβ-IR area in vessels (n=28). Data from individual human donor as well as group means and SEMs are shown. *p < 0.05, **p < 0.01, ***p < 0.001, ****p < 0.0001, by one-way or two-way ANOVA test with Sidak's post-hoc multiple comparison test. Fold changes are shown in red.

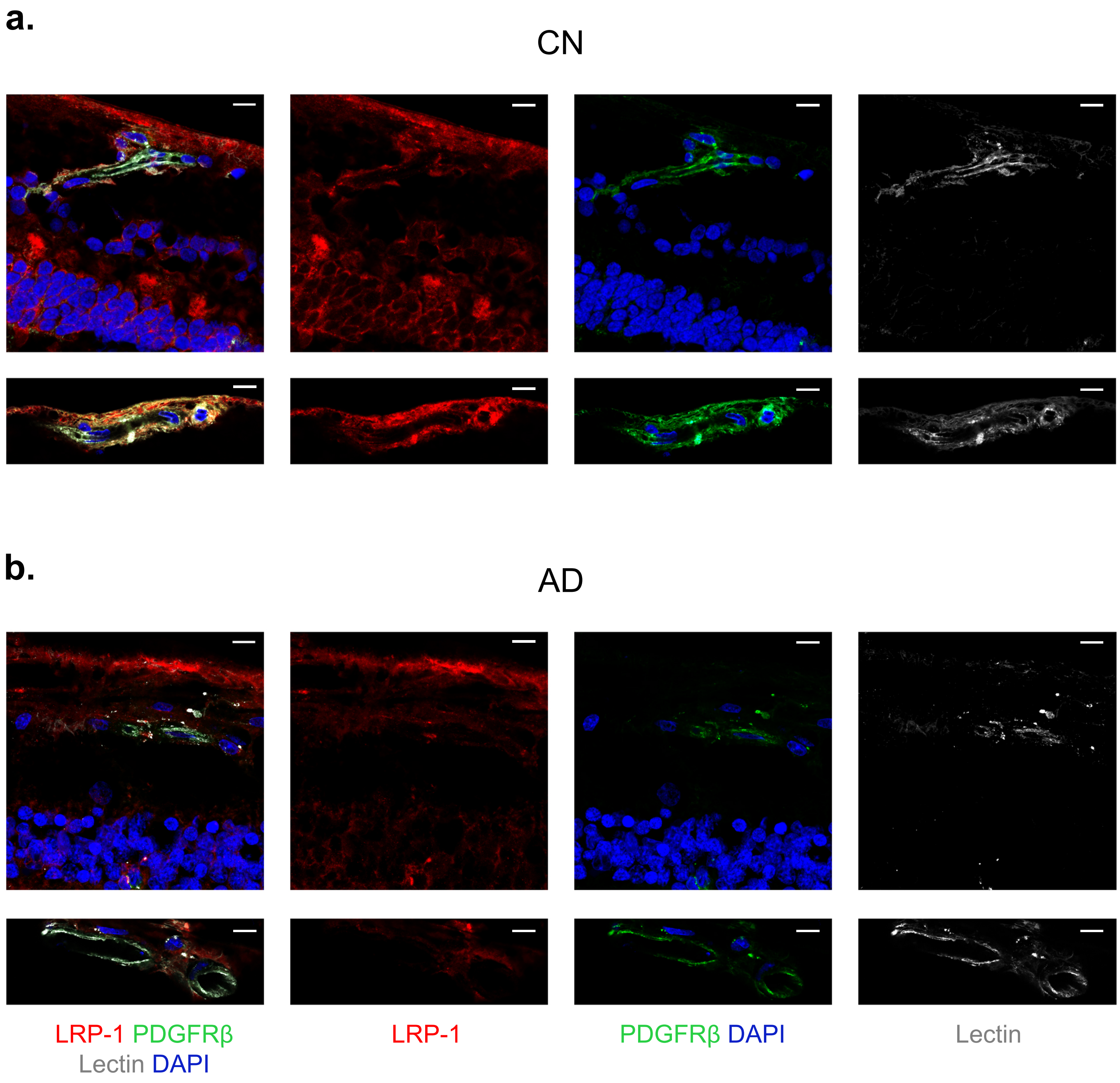

Supplementary Figure 12. Extended representative images for LRP-1 in figure 6. **a-b**. Merged and separate channels of representative fluorescent images for LRP-1 (red), PDGFRβ (green), lectin (glycoprotein in blood vessel, white) and DAPI (nuclei, blue) in paraffin-embedded retinal cross-sections isolated from age and sex-matched human donors with AD or cognitively normal (CN). **a**. CN and **b**. AD are shown. Scale bars=10μm.

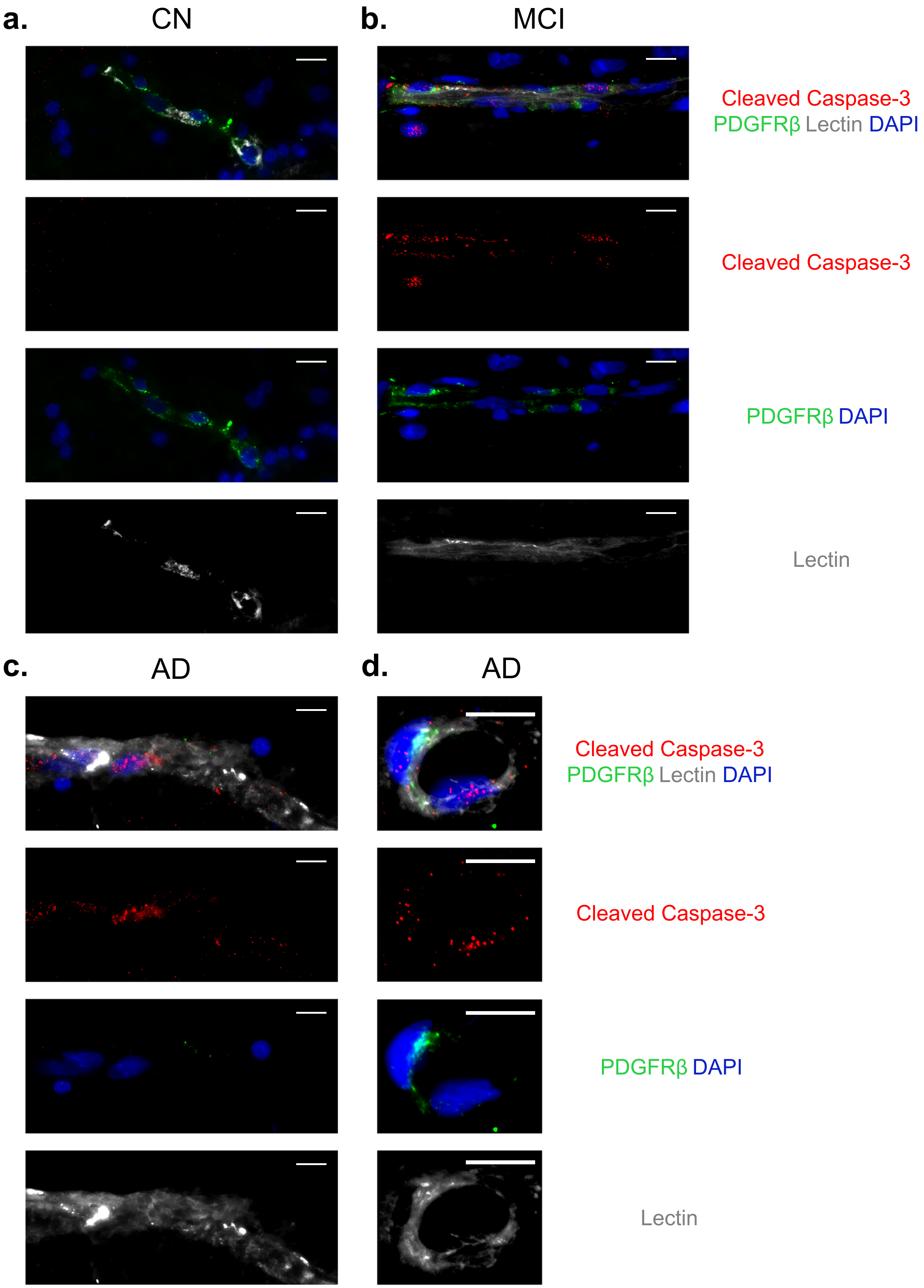

Supplementary Figure 13. Extended representative images for cleaved caspase-3 in figure 6. **a-d**. Merged and separate channels of fluorescent images for cleaved caspase-3 (red), PDGFRβ (green), lectin (glycoprotein in blood vessel, white) and DAPI (nuclei, blue) in paraffin-embedded retinal cross-sections isolated from age and sex-matched human donors with AD, MCI or cognitively normal (CN). **a**. CN, **b**. MCI and **c-d**. AD are shown. Scale bars=10μm.

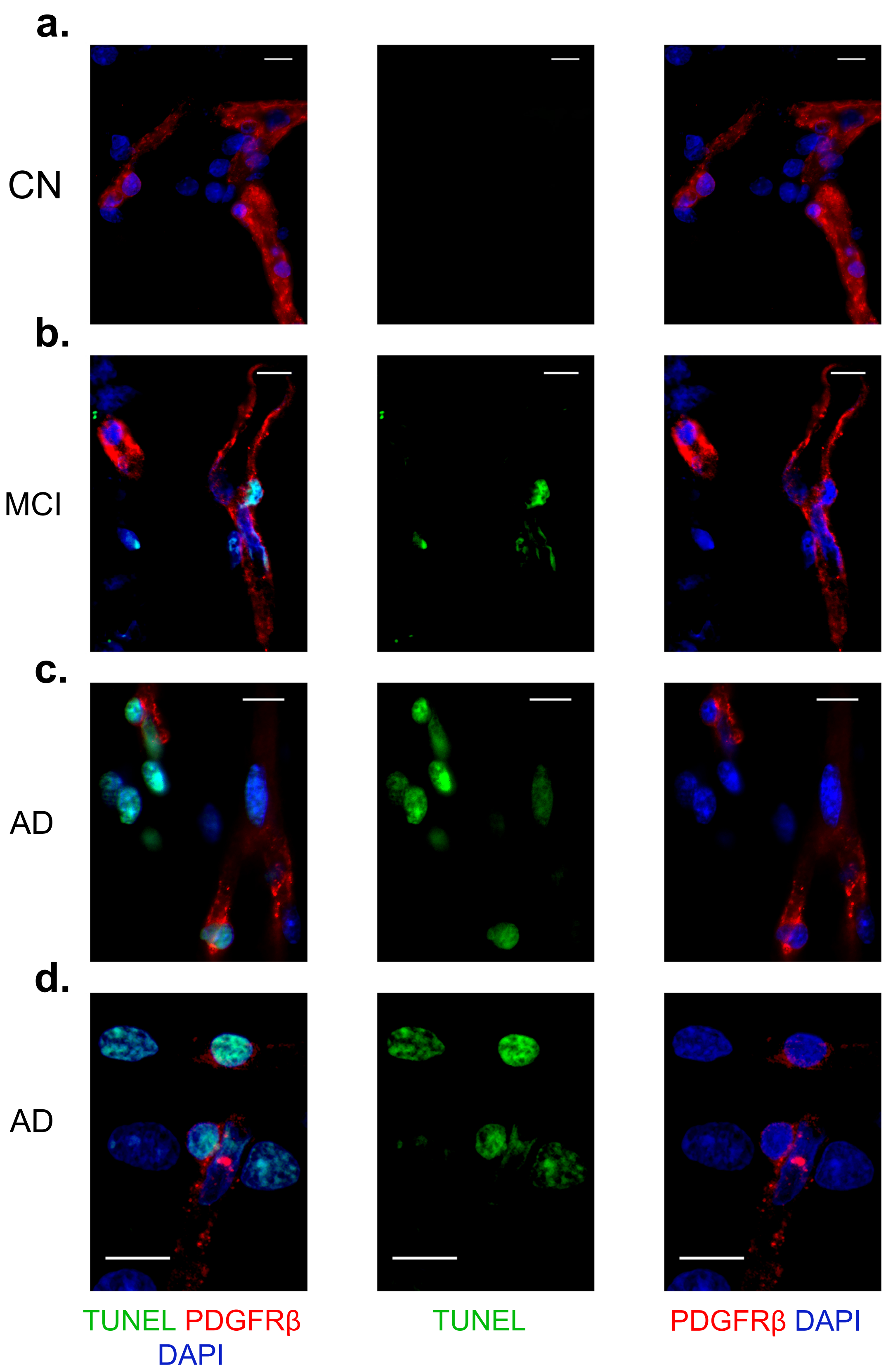

Supplementary Figure 14. Extended representative images for TUNEL staining in figure 6. **a-d**. Merged and separate channels of fluorescent images for TUNEL (green), PDGFRβ (red), and DAPI (nuclei, blue) in paraffin-embedded retinal cross-sections isolated from age and sex-matched human donors with AD, MCI or cognitively normal (CN). **a**. CN, **b**. MCI and **c-d**. AD are shown. Scale bars=10μm.

Table S1. Demographic data on human eye donors evaluated by retinal cross-section.

	CN	MCI	AD	F	P
**No. of Subjects**	14	11	21	-	-
**(n=46)**	(9F, 5M)	(5F, 6M)	(13F, 8M)		
**Age ± SD**	79.14 ± 10.5	87.09 ± 5.4	81.81 ± 14.9	1.404	0.2567
**[Years]**	F: 79.78 ± 12.3	F, 90.2 ± 3.6	F, 85.61 ± 12.6		
	M: 78.0 ± 7.3	M, 84.5 ± 5.5	M, 67.2 ± 16.7		
**Race**	13C (92.9%)	7C (81.8%)	16C (76.2%)	-	-
**(%)**	1B (7.1%)	1H (9.1%)	1B (4.8%)		
		1B (9.1%)	4A (19%)		
**PMI** **[Hours]**	7.5 ± 2.3	9.5 ± 5.0	7.6 ± 3.7	1.120	0.3355

CN cognitively normal; MCI, mild cognitive impairment; AD, Alzheimer's disease; F, female, M, male; SD, standard deviation; C, Caucasian; B, Black; H, Hispanic; A, Asian, UK Unknown; PMI, post-mortem interval; Values are presented as mean ± SD. F and P values were determined by one-way ANOVA with Sidak's multiple comparison test.

Table S2. Neuropathological evaluation in a subset of human donors evaluated by retinal cross-section.

Brain Scores	CN* (n=1)	MCI (n=7)	AD (n=17)
CAA	1	0.7 ± 0.97	1.3 ± 0.75
Aβ Plaque	0.545	2.11 ± 0.77	2.8 ± 0.93
Neurofibrillary Tangle	0.98	1.46 ± 0.99	2.5 ± 1.3
Neuropil Thread	0.86	1.1 ± 0.93	1.13 ± 1.2
Atrophy	0.8	1.09 ± 1.08	2.05 ± 1.2

CN cognitively normal; MCI, mild cognitive impairment; AD, Alzheimer's disease; A neuropathological score from one CN donor. CAA, cerebral amyloid angiopathy.

Table S3. Multiple correlation analysis between % retinal PDGFβ area in vessels and neuropathological parameters

		Neuritic Plaques	Immature Plaques	Diffuse Plaque	NFTs (Silver)	Neuropil Threads
All Brain	*r*	**- 0.50**	-0.36	-0.45	- 0.07	- 0.03
	P	***0.0264***	*0.1224*	*0.0443*	*0.7801*	*0.9022*
Hippocampus	*r*	**- 0.61**	-0.40	-0.38	- 0.17	0.12
	P	***0.0046***	*0.0802*	*0.0986*	*0.4622*	*0.6184*
Entorhinal Cortex	*r*	**0.26**	-0.01	0.35	0.15	- 0.23
	P	***0.0433***	*0.2598*	*0.3403*	*0.4902*	*0.5154*
Frontal Cortex	*r*	-0.01	-0.30	-0.04	-0.38	- 0.34
	P	*0.9543*	*0.1990*	*0.8586*	*0.1027*	*0.1396*
Temporal Cortex	*r*	-0.06	-0.33	-0.35	-0.09	-0.18
	P	*0.8066*	*0.1551*	*0.1265*	*0.7234*	*0.4504*
Parietal Cortex	*r*	0.05	0.01	-0.28	-0.33	-0.39
	P	*0.8431*	*0.9769*	*0.2280*	*0.1511*	*0.0938*
PV. Ctx. A-17	*r*	-0.32	-0.35	-0.46	-0.25	**-0.55**
	P	*0.2134*	*0.1540*	*0.0568*	*0.3412*	***0.0175***
VA. Ctx. A-18	*r*	**-0.74**	-0.30	**-0.58**	-0.39	-0.46
	P	***0.0015***	*0.2598*	***0.0192***	*0.1457*	*0.0872*

Correlations between retinal % area of PDGFβ in vessels and the corresponding neuropathological measurements: neuritic plaques, immature plaques, diffuse plaques, neurofibrillary tangles (NFTs; by Gallyas Silver stain), neuropil threads by sliver stain. Scores are given as: 0 = None, 1 = Sparse (-5), 3 = Moderate (6-20), 5 = Frequent (21-30 or above) based on pathological reports. Analysis was performed for mean of all brain regions and separated for each brain region. Sample size: n=14 for AD, n=5 for MCI, n=1 for CN. Statistical significance *P* is < 0.05 indicated in bold red color. Pearson's r correlations analysis was applied to determine relationships; PV – primary visual; VA – visual association; Ctx – cortex.

Table S4. Multiple correlation analysis between % retinal $A\beta_{40}$ area in vessels and neuropathological parameters

		Neuritic Plaques	Immature Plaques	Diffuse Plaques	NFTs (Silver)	Neuropil Threads
All Brain	*r*	**0.55**	0.44	0.16	0.24	**0.58**
	P	***0.0492***	*0.1292*	*0.6122*	*0.4278*	***0.0375***
Hippocampus	*r*	0.41	0.22	0.08	0.37	0.48
	P	*0.1649*	*0.4702*	*0.7984*	*0.211*	*0.0967*
Entorhinal Cortex	*r*	**0.77**	0.47	0.2	0.33	0.31
	P	***0.0023***	*0.1012*	*0.505*	*0.2755*	*0.3018*
Frontal Cortex	*r*	0.05	0.53	-0.06	0.40	0.53
	P	*0.8747*	*0.0638*	*0.8475*	*0.1777*	*0.0616*
Temporal Cortex	*r*	0.00	0.23	0.02	0.06	0.57
	P	*0.9878*	*0.4551*	*0.9574*	*0.8534*	*0.0507*
Parietal Cortex	*r*	0.02	-0.09	0.15	0.14	0.38
	P	*0.941*	*0.7627*	*0.6274*	*0.6522*	*0.2022*
PV. Ctx. A-17	*r*	0.18	**0.6**	0.21	-0.19	**0.64**
	P	*0.5929*	***0.0373***	*0.5179*	*0.5798*	***0.0347***
VA. Ctx. A-18	*r*	0.54	0.53	-0.09	-0.03	**0.84**
	P	*0.1377*	*0.1137*	*0.8081*	*0.93*	***0.0042***

Correlations between retinal % area of $A\beta_{40}$ in vessels and the corresponding neuropathological measurements: neuritic plaques, immature plaques, diffuse plaques, neurofibrillary tangles (NFTs; by Gallyas Silver stain), neuropil threads by sliver stain. Scores are given as: 0 = None, 1 = Sparse (-5), 3 = Moderate (6-20), 5 = Frequent (21-30 or above) based on pathological reports. Analysis was performed for mean of all brain regions and separated for each brain region. Sample size: n=8 for AD, n=3 for MCI, n=1 for CN patients. Statistical significance *P* is < 0.05 indicated in bold red color. Pearson's r correlations analysis was applied to determine relationships; PV – primary visual; VA – visual association; Ctx – cortex.

Table S5. Correlation between % PDGFRβ immunoreactive area per retinal subregions and MMSE scores.

Retinal Regions	Total	ST	TI	IN	NS	Superior	Inferior	Nasal	Temporal
r	0.77	0.89	0.82	0.71	0.92	0.92	0.71	0.73	0.89
P	0.0156	0.016	0.1825	0.0499	0.0271	0.0036	0.0486	0.0411	0.0171
N	9	6	4	8	5	7	8	8	6

Total; total retinal average; ST, superiortemporal; TI, temporalinferior; IN, inferioirnasal; NS, nasalsuperior; Superior, mean of ST and NS values; Inferior, mean of TI and IN values; Nasal, mean of IN and NS values; Temporal, mean of ST and TI values. N, number of pairs. Statistical significance P is < 0.05. Pearson's r correlations analysis was applied to determine relationships.

Table S6. Correlation between $A\beta_{40}$ burden per retinal subregions and MMSE cognitive scores.

Retinal Regions	Total	ST	TI	IN	NS	Superior	Inferior	Nasal	Temporal
r	-0.57	-0.71	N/A	-0.65	N/A	-0.73	-0.67	-0.66	-0.75
P	0.0827	0.0737	N/A	0.1107	N/A	0.0625	0.099	0.1062	0.0539
n	10	7	N/A	7	N/A	7	7	7	7

Total; total retinal average; ST, superiortemporal; TI, temporalinferior; IN, inferioirnasal; NS, nasalsuperior; Superior, mean of ST and NS values; Inferior, mean of TI and IN values; Nasal, mean of IN and NS values; Temporal, mean of ST and TI values. N, number of pairs. N/A, not applicable. Statistical significance P is < 0.05. Pearson's r correlations analysis was applied to determine relationships.
